# Supplementary material for: Residential area deprivation and risk of subsequent hospital admission in a British population: the EPIC-Norfolk cohort
Source: BMJ Open. 2019 Dec 16;9(12):e031251. doi: 10.1136/bmjopen-2019-031251 (PMC6937051; doi:10.1136/bmjopen-2019-031251)
Supplement: Supplementary data [file bmjopen-2019-031251supp001.pdf]

Supplementary Table S1 | Multivariable logistic regression of risk factors for any hospital admissions, ≥7 hospital admissions and >20 days of hospital stay from 1999 to 2018 in 24 977 men and women. Townsend index components using the UK 1991 census

	All subjects OR (95% CI)	p value	Men OR (95% CI)	p value	Women OR (95% CI)	p value
**Outcome of any hospital admissions**						
Male sex	1.13 (1.04–1.23)	0.006				
Age per 10 years	2.08 (1.98–2.20)	< 0.001	2.15 (1.99–2.34)	< 0.001	2.03 (1.89–2.17)	< 0.001
Unemployment Z-Score >0	1.06 (0.92–1.21)	0.426	1.06 (0.87–1.31)	0.560	1.05 (0.88–1.26)	0.604
Households with no car Z-Score >0	1.05 (0.89–1.23)	0.568	0.91 (0.72–1.16)	0.459	1.16 (0.94–1.44)	0.161
Households not owner-occupied Z-Score >0	1.03 (0.90–1.19)	0.625	0.99 (0.81–1.22)	0.948	1.07 (0.89–1.28)	0.479
Household overcrowding Z-Score >0	0.98 (0.88–1.10)	0.760	1.00 (0.84–1.19)	0.983	0.97 (0.84–1.13)	0.717
Manual social class	1.16 (1.06–1.27)	0.001	1.25 (1.08–1.43)	0.002	1.11 (0.98–1.25)	0.106
Low education level	1.35 (1.22–1.50)	< 0.001	1.21 (1.03–1.43)	0.026	1.45 (1.27–1.66)	< 0.001
Current smoker	1.21 (1.06–1.38)	0.006	1.15 (0.94–1.41)	0.174	1.26 (1.05–1.51)	0.013
BMI>30 kg/m^2	1.28 (1.12–1.46)	< 0.001	1.19 (0.97–1.47)	0.095	1.34 (1.13–1.59)	< 0.001
**Outcome of 7 or more hospital admissions**						
Male sex	1.23 (1.16–1.30)	< 0.001				
Age per 10 years	1.49 (1.44–1.54)	< 0.001	1.50 (1.43–1.57)	< 0.001	1.48 (1.42–1.55)	< 0.001
Unemployment Z-Score >0	1.18 (1.08–1.28)	< 0.001	1.17 (1.04–1.33)	0.012	1.18 (1.06–1.33)	0.004
Households with no car Z-Score >0	0.98 (0.88–1.08)	0.641	0.92 (0.79–1.06)	0.253	1.03 (0.90–1.17)	0.692
Households not owner-occupied Z-Score >0	0.93 (0.86–1.02)	0.134	0.91 (0.80–1.04)	0.163	0.95 (0.85–1.08)	0.447
Household overcrowding Z-Score >0	1.08 (1.00–1.16)	0.044	1.07 (0.96–1.19)	0.207	1.08 (0.98–1.20)	0.112
Manual social class	1.15 (1.08–1.22)	< 0.001	1.16 (1.06–1.26)	< 0.001	1.14 (1.06–1.24)	0.001
Low education level	1.14 (1.07–1.21)	< 0.001	1.12 (1.02–1.23)	0.014	1.15 (1.06–1.25)	< 0.001
Current smoker	1.34 (1.23–1.46)	< 0.001	1.25 (1.10–1.41)	< 0.001	1.44 (1.28–1.61)	< 0.001
BMI>30 kg/m^2	1.38 (1.28–1.49)	< 0.001	1.39 (1.24–1.56)	< 0.001	1.38 (1.25–1.52)	< 0.001
**Outcome of more than 20 hospital days**						
Male sex	1.16 (1.10–1.23)	< 0.001				
Age per 10 years	2.68 (2.59–2.78)	< 0.001	2.62 (2.49–2.76)	< 0.001	2.73 (2.61–2.87)	< 0.001
Unemployment Z-Score >0	1.10 (1.01–1.20)	0.034	1.10 (0.96–1.25)	0.161	1.10 (0.98–1.24)	0.111
Households with no car Z-Score >0	1.00 (0.90–1.11)	0.979	0.95 (0.81–1.11)	0.501	1.04 (0.90–1.19)	0.599
Households not owner-occupied Z-Score >0	1.00 (0.92–1.10)	0.916	1.01 (0.88–1.16)	0.849	1.00 (0.88–1.13)	0.958
Household overcrowding Z-Score >0	1.07 (0.99–1.15)	0.100	1.03 (0.92–1.15)	0.622	1.10 (0.99–1.22)	0.071
Manual social class	1.16 (1.09–1.24)	< 0.001	1.19 (1.09–1.31)	< 0.001	1.14 (1.05–1.24)	0.003
Low education level	1.16 (1.09–1.24)	< 0.001	1.18 (1.07–1.30)	< 0.001	1.15 (1.05–1.25)	0.001
Current smoker	1.57 (1.44–1.72)	< 0.001	1.61 (1.41–1.83)	< 0.001	1.54 (1.37–1.74)	< 0.001
BMI>30 kg/m^2	1.61 (1.49–1.74)	< 0.001	1.58 (1.40–1.79)	< 0.001	1.63 (1.48–1.81)	< 0.001

Supplementary Table S2 | Multivariable logistic regression of Townsend Index and more than twenty hospital days in subgroups

	Townsend Index OR (95% CI)†
**Men and women**	
Men (n=11214)	1.01 (0.99–1.03)
Women (n=13763)	1.03 (1.01–1.05)
**By age above and below 65 years**	
Less than 65 years (n=17343)	1.03 (1.01–1.05)
65 years and above (n=7634)	1.01 (0.98–1.03)
**Manual and non-manual social class**	
Non-manual (n=14691)	1.02 (1.00–1.04)
Manual (n=9741)	1.03 (1.00–1.05)
**By level of education**	
Higher level (n=15841)	1.01 (0.99–1.03)
Lower level (n=9118)	1.03 (1.01–1.06)
**By smoking status**	
Former or never smoker (n=21864)	1.02 (1.01–1.04)
Current smoker (n=2895)	1.00 (0.97–1.04)
**By level of body mass index**	
BMI ≤ 30 kg/m² (n=21124)	1.02 (1.01–1.04)
BMI > 30 kg/m² (n=3800)	1.01 (0.98–1.04)
**Urban or rural home postcode**	
Urban (n=11214)	1.01 (1.00–1.03)
Rural (n=13763)	1.03 (1.01–1.06)
**Moved house between 2000 and 2014**	
Moved house (n=5355)	1.04 (1.01–1.07)
Did not move house (n=18728)	1.01 (1.00–1.03)

Logistic regression per unit Townsend Index. † Adjusted for age, current smoking, BMI (categories)
