# Supplementary material for: Residential area deprivation and risk of subsequent hospital admission in a British population: the EPIC-Norfolk cohort
Source: BMJ Open. 2019 Dec 16;9(12):e031251. doi: 10.1136/bmjopen-2019-031251 (PMC6937051; doi:10.1136/bmjopen-2019-031251)
Supplement: Author's manuscript [file bmjopen-2019-031251.draft_revisions.pdf]

BMJ Open is committed to open peer review. As part of this commitment we make the peer review history of every article we publish publicly available.

When an article is published we post the peer reviewers' comments and the authors' responses online. We also post the versions of the paper that were used during peer review. These are the versions that the peer review comments apply to.

The versions of the paper that follow are the versions that were submitted during the peer review process. They are not the versions of record or the final published versions. They should not be cited or distributed as the published version of this manuscript.

BMJ Open is an open access journal and the full, final, typeset and author-corrected version of record of the manuscript is available on our site with no access controls, subscription charges or pay-per-view fees (http://bmjopen.bmj.com).

If you have any questions on BMJ Open's open peer review process please email info.bmjopen@bmj.com

BMJ Open

Residential area deprivation predicts subsequent hospital admission over 19 years in a British population independently of social class and education status: the EPIC-Norfolk cohort

Journal:	*BMJ Open*
Manuscript ID	bmjopen-2019-031251
Article Type:	Research
Date Submitted by the Author:	24-Apr-2019
Complete List of Authors:	Luben, Robert; University of Cambridge, Department of Public Health and Primary Care<br>Hayat, Shabina; University of Cambridge, Department of Public Health & Primary Care<br>Khawaja, Anthony; Moorfields Eye Hospital, NIHR Biomedical Research Centre<br>Wareham, Nicholas; University of Cambridge School of Clinical Medicine, MRC Epidemiology Unit<br>Pharoah, Paul; University of Cambridge, Department of Public Health and Primary Care<br>Khaw, Kay-Tee; University of Cambridge, Clinical Medicine
Keywords:	EPIDEMIOLOGY, Health policy < HEALTH SERVICES ADMINISTRATION & MANAGEMENT, PUBLIC HEALTH, SOCIAL MEDICINE

SCHOLARONE™
Manuscripts

Residential area deprivation predicts subsequent hospital admission over 19 years in a British population independently of social class and education status: the EPIC-Norfolk cohort

Robert Luben[1], Shabina Hayat[1], Anthony Khawaja[2], Nicholas Wareham[3], Paul Pharoah[1] , Kay-Tee Khaw[1]

1 Department of Public Health and Primary Care, Institute of Public Health, University of Cambridge, Cambridge, United Kingdom
2 NIHR Biomedical Research Centre, Moorfields Eye Hospital NHS Foundation Trust and UCL Institute of Ophthalmology, London, UK
3 MRC Epidemiology Unit, University of Cambridge School of Clinical Medicine, Cambridge United Kingdom
Correspondence to: Robert Luben, Department of Public Health and Primary Care, Strangeways Research Laboratory, Worts Causeway, Cambridge, UK. Email: robert.luben@phpc.cam.ac.uk Telephone: +44 (0)1223 748691

Word count: 4,093

April 24, 2019

For peer review only

ABSTRACT

OBJECTIVES

To investigate whether residential area deprivation index predicts subsequent admissions to hospital and time spent in hospital independently of individual social class and lifestyle factors.

DESIGN

Prospective population based study.

SETTING

The EPIC-Norfolk Study.

PARTICIPANTS

11214 men and 13763 women in the general population, aged 40-79 years at recruitment (1993 to 1997), alive in 1999.

MAIN OUTCOME MEASURE

Total admissions to hospital and time spent in hospital during a 19 year time period (1999 to 2018).

RESULTS

Compared to those with residential Townsend Area Deprivation Index lower than the average for England and Wales, those with a higher than average deprivation index had a higher likelihood of spending more than twenty days in hospital multivariable adjusted odds ratio (OR) 1.18 (95% confidence interval (CI) 1.07 to 1.29) and having 7 or more admissions OR 1.11 (95% CI 1.02 to 1.22) after adjustment for age, sex, smoking status, education, social class, and body mass index. Occupational social class and educational attainment modified the association between area deprivation and hospitalisation; those with manual social class and lower education level were at greater risk of hospitalisation when living in an area with higher deprivation index ($p_{Interaction}$=0.02 and $p_{Interaction}$=0.02 respectively), while the risk for non-manual and more highly educated participants did not vary greatly by area of residence.

CONCLUSION

Residential area deprivation predicts future hospitalisations, time spent in hospital and number of admissions, independently of individual social class and education level and other behavioural factors. There are significant interactions such that residential area deprivation has greater impact in those with low education level or manual social class. Conversely, higher education level and social class mitigated the association of area deprivation with hospital usage.

For peer review only

STRENGTHS AND LIMITATIONS OF THIS STUDY

- This study is able to examine hospital activity using a prospective cohort design in a population of community-dwelling participants with clearly defined population denominators.
- It uses a large cohort of middle aged and older men and women with 19 years of follow-up time and detailed measurements of demographic and behavioural indicators.
- Both area based census measures and individual social class and education level from questionnaires are used.
- Differential misclassification in hospital use may be explained by early death rates.
- We were only able to examine National Health Service hospitals and not private clinics where study participants chose to pay for treatment.

For peer review only

Acknowledgements: The authors would like to thank the participants, general practitioners, and staff of EPIC-Norfolk.

Contributors: KTK,NW SH and RL were involved in the conception and design of the study. RL drafted the manuscript, with support from KTK and PP. AK contributed to data interpretation. RL was responsible for external data linkage. SH and RL contributed to data collection and acquisition. All authors read and critically revised the manuscript and approved the final manuscript. RL is the guarantor. The corresponding author attests that all listed authors meet authorship criteria and that no others meeting the criteria have been omitted.

Funding: EPIC-Norfolk infrastructure and core functions are supported by grants from the Medical Research Council (G0401527) and Cancer Research UK (C864/A8257).

Competing interests: All authors have completed the ICMJE uniform disclosure form at www.icmje.org/coi_disclosure.pdf and report: RL,SH,KTK and NW report grants from MRC and CRUK during the conduct of the study; no support from any organisation for the submitted work; no financial relationships with any organisations that might have an interest in the submitted work in the previous three years; no other relationships or activities that could appear to have influenced the submitted work.

Ethical approval: The work was approval by the East Norfolk and Waveney NHS research governance committee (2005EC07L) and the Norfolk research ethics committee (05/Q0101/191). All participants gave informed signed consent for study participation including access to medical records.

Data sharing: The authors will make the dataset available under a Data Transfer Agreement to any bona fide researcher who wishes to obtain the dataset in order to undertake a replication analysis. Although the dataset is anonymised, the breadth of the data included and the multiplicity of variables that are included in this analysis file as primary variables or confounding factors, means that provision of the dataset to other researchers without a Data Transfer Agreement would constitute a risk.

Transparency: The lead author (the manuscript's guarantor) affirms that the manuscript is an honest, accurate, and transparent account of the study being reported; that no important aspects of the study have been omitted; and that any discrepancies from the study as planned (and, if relevant, registered) have been explained.

Open access: This is an Open Access article distributed in accordance with the terms of the Creative Commons Attribution (CC BY 4.0) license, which permits others to distribute, remix, adapt and build upon this work, for commercial use, provided the original work is properly cited. See: http://creativecommons.org/licenses/by/4.0/

Patient and public involvement: The EPIC-Norfolk Study have an active Participants Advisory Panel which meets quarterly to advise on research protocols, suggest ideas and provide feedback on the research including proposed new studies and collaborations. All participants of the EPIC-Norfolk study are informed about the study through regular newsletters as well as public meetings. Information is also disseminated through local community talks in the Norfolk area and science festivals.

Exclusive licence: The Corresponding Author has the right to grant on behalf of all authors and does grant on behalf of all authors, a worldwide licence to the Publishers and its licensees in perpetuity, in all forms, formats and media (whether known now or created in the future), to i) publish, reproduce, distribute, display and store the Contribution, ii) translate the Contribution into other languages, create adaptations, reprints, include within collections and create summaries, extracts and/or, abstracts of the Contribution, iii) create any other derivative work(s) based on the Contribution, iv) to exploit all subsidiary rights in the Contribution, v) the inclusion of electronic links from the Contribution to third party material where-ever it may be located; and, vi)

licence any third party to do any or all of the above.

The Corresponding Author has the right to grant on behalf of all authors and does grant on behalf of all authors, an exclusive licence (or non exclusive for government employees) on a worldwide basis to the BMJ Publishing Group Ltd to permit this article (if accepted) to be published in BMJ editions and any other BMJPGL products and sublicences such use and exploit all subsidiary rights, as set out in our licence.

For peer review only

Introduction

The considerable differences in mortality by social class are well documented [1–4] with those in higher social classes having a typical life expectancy several years longer than those with the lowest. Similarly, life expectancy and health expectancy varies between UK cities and regions with large variations in expected years of life in good health. [5,6] Despite increasing overall life expectancy, inequality remains with lower life and health expectancy observed more often in disadvantaged groups. While lifestyle factors may account for some part of this, the reported differences in death rates cannot be explained by individual behaviour alone. [7,8] Material deprivation was defined by Townsend as 'a state of observable and demonstrable disadvantage relative to the local community or wider society … to which an individual, family or group belongs'. Deprivation indices use factors such as unemployment, the standard of housing, overcrowding and rates of car ownership which together can assess the level of deprivation within a neighbourhood. [9]

Socioeconomic determinants of hospitalisation have been examined using individual level exposures such as occupational social class, income and education and at area level using various deprivation indices. Individual occupational social class, income and level of education have all been reported to be associated with chronic disease risk. [10] Area based deprivation measures, available routinely in the UK using postal code linkage, have also reported associations with hospital usage. [11–15] However, the participants in such studies are often limited to those attending hospital and so a suitable population denominator is lacking. Studies reporting health associations for both individual and area measures are less common [16–18] with few if any examining the independent association of residential area deprivation on subsequent hospital admission.

Hospitalisation can be measured using the frequency of admission or the length of stay. When measured over a period of time, the outcome represents burden of resources that might be attributable to a population. The UK National Health Service is free at the point of use and consequently should provide equitable healthcare. We previously reported that a range of simple demographic and behavioural indicators are related to the future probability of cumulative hospital admissions and bed days. [19] Increasing age and male sex and the modifiable factors current cigarette smoking, body mass index (BMI) >30 kg/m^2, manual social class and low education level were all associated with higher future hospital usage over a ten year period. In this paper we examine residential area deprivation using the Townsend deprivation index at enumeration district level from the UK 1991 census, just prior to the recruitment of the cohort with subsequent hospital usage over a 19 year period. We explore the independent contribution of residential area deprivation in men and women participants of the EPIC-Norfolk study and its association with future hospitalisation after allowing for the individual level factors previously shown be associated.

Methods

We used data collected as part of the European Prospective Investigation into Cancer in Norfolk (EPIC-Norfolk), a general population cohort.

Study design

EPIC-Norfolk is a cohort of men and women living in Norfolk. Recruitment took place between 1993 and 1997 at 35 general practices with invitations sent to all those registered with the practices aged 40–79 years. The design and recruitment of the study has been previously described in detail. [20,21] Briefly, a total of 77630 invitations were sent; 30445 (40%) consented to participate of whom 25639 men and women, completed a lifestyle

questionnaire and attended a health examination.

Residential area deprivation score for participants

The Townsend Index is an area deprivation measurement calculated using four components: the percentage unemployed of economically active residents over 16 years old, the percentage of households with no car, the percentage of households not owner occupied, and the percentage of households with more than one person per room. These are respectively: a measure of lack of material resources and insecurity, a proxy for current income, a proxy for current wealth, and a measure of material living conditions. The index used in this study was constructed using data collected at the 1991 UK census, which takes place every ten years, with each Townsend component calculated at Enumeration District (ED), a small area containing an average 175 households used both as output areas and for data collection. Townsend components were then standardised as Z scores at ED level for England and Wales. Study participants were linked to an ED using their home postcode at the end of recruitment in the year 2000. The link was then used to establish a residential Townsend Index for each individual.

Covariables

Participants' height and weight were measured in light clothing without shoes by trained nurses in a clinic setting as part of a health examination between 1993 and 1997. Height was measured to the nearest 0.1cm using a stadiometer (Chasemores, UK) and weight to the nearest 0.1kg. BMI was calculated using measured weight in kilograms divided by measured height^2 in square metres.

Participants completed a lifestyle questionnaire which included questions about their and their partner's current and past employment. Occupational social class was defined according to the Registrar General's classification [22] and dichotomised into non-manual and manual social classes. Professional, managerial and technical and non-manual skilled occupations (codes I, II and IIIa respectively) were classed as non-manual while manual skilled, partly skilled and unskilled (codes IIIb, IV and V respectively) were classed as manual. Social class for men used (in order of priority) their own current employment, own past employment, partner's current employment or partner's past employment according to whether a social class classification could be defined for a given occupation type. Similarly, social class for women used (in order of priority) their partner's current employment, partner's past employment, own current employment, own past employment. The use of partner's social class for women in the EPIC-Norfolk cohort born between 1918 and 1948 has been previously discussed. [10]

The question "Do you have any of the following qualifications" together with a list of common UK qualifications was used to establish educational attainment. Participants were categorised according to the highest qualification they attained: those with no formal qualifications, those with formal qualifications usually associated with completing school aged between 16 and 18 years, and those with degree level qualifications.

Smoking status was derived from two questions: "Have you ever smoked as much as one cigarette a day for as long as a year" and "Do you smoke cigarettes now". The responses to both questions were "yes" or "no" and participants were asked to leave the second question blank if they answered "no" to the first.

Travel time and travel distance between participants home postcode and the Norfolk and Norwich University Hospitals NHS Foundation Trust was calculated using the Open Source Routing Machine (OSRM) [23] which calculates the shortest path between two points over the road network. Postcode of home residence was used to establish if a participant had moved house over the follow-up period. It was available at two points in time: in the year 2000 and the year 2014. Participants who postcode or house location remained unchanged over the period were classified as not moving house. Urban and rural categories were established using the 1991 census.

For peer review only

Ascertainment of hospital usage and mortality through record linkage

Details of linkage of the EPIC-Norfolk cohort participants to hospital records have been previously reported. [19] Briefly, linkage using unique NHS numbers was performed between 1999 and 2018 to databases maintained by the East Norfolk Primary Health Care Trust and to national databases held by NHS Digital. [24] All hospital activity for EPIC-Norfolk participants was captured wherever they were treated in England and Wales. Hospital episode statistics (HES) records which included admission and discharge dates were used to calculate time in hospital and numbers of admissions. Contiguous admissions were merged and counted as a single admission.

Statistical analysis

For the current analyses, we excluded the 625 men and women from the baseline cohort who died before 1999. A further 37 who did not have a valid UK postcode were excluded leaving 24977. Dichotomous variables were created for the three socioeconomic status variables. Occupational social class was categorised into non-manual and manual: social classes I, II, and III non-manual were classified as 'non-manual', while social classes III manual, IV, and V were classified as 'manual'. Educational level was categorised into 'Higher level' (which includes those with qualifications at secondary level or above) and 'Lower level' (those with no qualification). Townsend deprivation index was divided into quintiles. Lower Townsend scores correspond to lower levels of deprivation. Quintiles 1-4 are all below zero and hence below (less deprived than) the national average for England and Wales. Quintile 5 (-0.64, 6.99] corresponds to Townsend scores close to or above the national average (more deprived). Overall Townsend score and components were also dichotomised with scores below zero defined as 'less deprived' and scores above 0 as 'more deprived'. Hospital admissions were categorised into five groups: 0, 1, 2–3, 4–6, and ≥7 while time in hospital was divided into categories: None, Day case, 2–5 days, 6–20 days, and >20 days. The cut-points were chosen to be consistent with earlier work. A dichotomous urban/rural variable was defined with "urban" and "urban sparse" as urban and "town", "village" or "hamlet" as rural. The numbers of individuals with missing values for covariables were:53 BMI, 218 smoking status, 545 social class, 18 education level. Three dichotomous outcome categories were calculated: any hospital admissions (vs no admissions), 7 or more admissions (vs fewer than 7) using total admissions, and greater than 20 hospital days (vs 20 or fewer) using total bed days (overnight stays) and day cases. All analyses were performed using the R statistical language (R Foundation for Statistical Computing, Vienna, Austria version 3.5.3 with packages knitr, Gmisc, ggplot2, and dplyr).

Results

Table 1 shows descriptive characteristics by quintiles of residential Townsend Index for 11214 men and 13763 women. The majority (n=20996) of study participants had deprivation index below zero while n=3981, approximately corresponding to those in quintile 5, had levels above the national average. Participants in quintile 5 were much more likely to live in an urban setting (70.2%) while those in quintiles 2,3 and 4 were more likely to live in a rural location. Travel distance was lowest for participants in quintile 1 and 5, perhaps due to a higher proportion living in cities and travel times followed a similar pattern. Participants in quintile 5 were the most likely to move house (26.1% between 2000 and 2014). Hospital admissions and time in hospital are shown for both the full cohort and restricted to those who attended hospital. 35.0% of study participants had no admissions over the 19 years from 1999 to 2018.

Table 1 | Descriptive characteristics by quintiles of Townsend Index

	Total	Quintile 1 [-6.74,-3.81]	Quintile 2 [-3.81,-2.94]	Quintile 3 [-2.94,-2.09]	Quintile 4 [-2.09,-0.64]	Quintile 5 (-0.64, 6.99]	P-value
**Sex [n (%)]**							
Men	11214 (44.9)	2271 (45.2)	2262 (45.4)	2280 (45.2)	2226 (45.0)	2175 (43.7)	0.41

	Total	Quintile 1 [-6.74,-3.81]	Quintile 2 (-3.81,-2.94]	Quintile 3 (-2.94,-2.09]	Quintile 4 (-2.09,-0.64]	Quintile 5 (-0.64, 6.99]	P-value
Women	13763 (55.1)	2752 (54.8)	2723 (54.6)	2760 (54.8)	2722 (55.0)	2806 (56.3)	
**Age, years**							
Mean ±SD	59.0 ±9.3	58.8 ±9.0	59.0 ±9.2	58.8 ±9.2	59.2 ±9.4	59.4 ±9.5	0.002
**Body mass index, kg/m²**							
Mean ±SD	26.4 ±3.9	26.1 ±3.8	26.3 ±3.8	26.4 ±3.9	26.5 ±4.0	26.5 ±4.1	< 0.001
**Cigarette smoking [n (%)]**							
Current	2895 (11.7)	457 (9.2)	501 (10.1)	569 (11.4)	575 (11.7)	793 (16.1)	< 0.001
Former	10411 (42.0)	2033 (40.7)	2083 (42.1)	2044 (41.0)	2132 (43.4)	2119 (43.1)	
Never	11453 (46.3)	2502 (50.1)	2361 (47.7)	2378 (47.6)	2203 (44.9)	2009 (40.8)	
**Social class dichotomised [n (%)]**							
Non-manual	14691 (60.1)	3336 (67.4)	3170 (64.8)	2950 (59.8)	2840 (58.9)	2395 (49.5)	< 0.001
Manual	9741 (39.9)	1610 (32.6)	1722 (35.2)	1985 (40.2)	1982 (41.1)	2442 (50.5)	
**Level of education [n (%)]**							
Higher level	15841 (63.5)	3439 (68.5)	3373 (67.7)	3218 (63.9)	3084 (62.4)	2727 (54.8)	< 0.001
Lower level	9118 (36.5)	1584 (31.5)	1611 (32.3)	1819 (36.1)	1858 (37.6)	2246 (45.2)	
**Travel distance to hospital, km**							
Mean ±SD	20.4 ±13.1	16.5 ±11.3	20.6 ±12.1	22.0 ±12.2	25.2 ±13.2	17.5 ±14.5	< 0.001
**Travel time to hospital, minutes**							
Mean ±SD	20.8 ±10.3	18.0 ±8.9	20.8 ±9.5	21.9 ±9.4	24.4 ±10.6	19.0 ±11.6	< 0.001
**Urban or rural location [n (%)]**							
Urban	11214 (44.9)	2500 (49.8)	1832 (36.8)	1810 (35.9)	1575 (31.8)	3497 (70.2)	< 0.001
Rural	13763 (55.1)	2523 (50.2)	3153 (63.2)	3230 (64.1)	3373 (68.2)	1484 (29.8)	
**Moved house between 2000 and 2014 [n (%)]**							
Moved house	5355 (22.2)	963 (19.8)	972 (20.4)	1091 (22.4)	1060 (22.4)	1269 (26.1)	< 0.001
Did not move house	18728 (77.8)	3903 (80.2)	3799 (79.6)	3774 (77.6)	3662 (77.6)	3590 (73.9)	
**Deaths prior to March 2018 [n (%)]**							
Dead	8727 (35.0)	1630 (32.5)	1704 (34.3)	1703 (33.9)	1781 (36.1)	1909 (38.4)	< 0.001
Alive	16198 (65.0)	3386 (67.5)	3270 (65.7)	3327 (66.1)	3155 (63.9)	3060 (61.6)	
**Hospital activity 1999-2018**							
No admissions	2628 (10.5)	543 (10.8)	528 (10.6)	539 (10.7)	559 (11.3)	459 (9.2)	0.011
One or more admissions	22316 (89.5)	4476 (89.2)	4449 (89.4)	4494 (89.3)	4383 (88.7)	4514 (90.8)	
**Time spent in hospital 1999-2018**							
Full cohort 1999-2018, mean ±SD	32.8 ±63.0	30.5 ±54.9	33.0 ±69.0	31.2 ±57.2	32.5 ±62.9	37.0 ±69.6	< 0.001
Hospital attenders 1999-2018, mean ±SD	36.7 ±65.6	34.2 ±57.0	36.9 ±72.0	34.9 ±59.5	36.6 ±65.7	40.8 ±72.0	< 0.001
**Number of inpatient admissions 1999-2018**							
Full cohort 1999-2018, mean ±SD	7.5 ±26.0	7.3 ±22.8	8.2 ±36.1	7.6 ±30.8	6.7 ±11.7	7.6 ±21.4	0.073
Hospital attenders 1999-2018, mean ±SD	8.4 ±27.3	8.2 ±24.0	9.2 ±38.1	8.6 ±32.5	7.6 ±12.1	8.4 ±22.3	0.095

Round brackets in intervals denote strict inequalities; square brackets denote non-strict inequalities

For peer review only

Table 2 shows the multivariable logistic regression for quintiles of Townsend Index and three outcomes: any hospital admissions, ≥7 hospital admissions, and >20 days of hospital stay between 1999 to 2018. Model 1 is adjusted for age and sex while model 2 is additionally adjusted for manual social class, lower education level, current cigarette smoking and body mass index >30 kg/m2. Additionally, each model is repeated in the subset of participants who survived to the end of the follow-up period. Compared to those with Townsend Deprivation Index quintiles 1 to 4 (lower than the average for England and Wales), those with a deprivation index in quintile 5 had a higher risk of spending more than 20 days in hospital multivariable adjusted odds ratio (OR) 1.18 (95% confidence interval (CI) 1.07–1.29) and for 7 or more hospital admissions OR 1.11 (95% CI 1.02–1.22), but there was no association for any hospital admission. The multivariable adjusted p-value for trend across quintiles of Townsend Index was 0.001 for more than 20 hospital days and 0.107 for 7 or more admissions. Associations in the subset of participants surviving to March 2018 (n=16198) were higher than those for the full cohort.

Table 2 | Multivariable logistic regression of risk factors by quintiles of Townsend Index for any hospital admissions, ≥7 hospital admissions and >20 days of hospital stay from 1999 to 2018 in 24977 men and women and in a subset of 16198 men and women alive in March 2018)

	Quintile 1 [-6.74,-3.81]	Quintile 2 (-3.81,-2.94]	Quintile 3 (-2.94,-2.09]	Quintile 4 (-2.09,-0.64]	Quintile 5 (-0.64, 6.99]	p (trend)
**Outcome of any hospital admissions**						
Model 1	1.00	1.02 (0.89–1.16)	1.01 (0.89–1.15)	0.93 (0.82–1.05)	1.17 (1.02–1.34)	0.175
Model 1†	1.00	1.05 (0.91–1.21)	1.03 (0.89–1.18)	0.95 (0.83–1.09)	1.25 (1.08–1.45)	0.056
Model 2	1.00	1.00 (0.88–1.14)	1.00 (0.87–1.14)	0.90 (0.79–1.03)	1.09 (0.95–1.26)	0.731
Model 2†	1.00	1.04 (0.90–1.20)	1.01 (0.88–1.16)	0.91 (0.79–1.05)	1.18 (1.01–1.37)	0.341
**Outcome of 7 or more hospital admissions**						
Model 1	1.00	1.08 (0.99–1.18)	1.09 (1.00–1.18)	1.05 (0.97–1.15)	1.20 (1.10–1.31)	< 0.001
Model 1†	1.00	1.06 (0.95–1.19)	1.15 (1.03–1.29)	1.15 (1.03–1.29)	1.39 (1.25–1.56)	< 0.001
Model 2	1.00	1.07 (0.98–1.16)	1.05 (0.96–1.15)	1.01 (0.92–1.10)	1.11 (1.02–1.22)	0.107
Model 2†	1.00	1.05 (0.93–1.17)	1.11 (0.99–1.24)	1.09 (0.97–1.22)	1.28 (1.14–1.43)	< 0.001
**Outcome of more than 20 hospital days**						
Model 1	1.00	1.09 (1.00–1.19)	1.10 (1.00–1.20)	1.14 (1.04–1.24)	1.27 (1.17–1.39)	< 0.001
Model 1†	1.00	1.08 (0.96–1.22)	1.15 (1.02–1.30)	1.23 (1.09–1.40)	1.45 (1.28–1.64)	< 0.001
Model 2	1.00	1.08 (0.99–1.18)	1.05 (0.96–1.15)	1.09 (1.00–1.19)	1.18 (1.07–1.29)	0.001
Model 2†	1.00	1.06 (0.94–1.21)	1.09 (0.96–1.23)	1.16 (1.03–1.32)	1.34 (1.18–1.51)	< 0.001

Model 1: Adjusted for age and sex. Model 2: Adjusted for age, sex, manual social class, low education, current cigarette smoker, body mass index > 30kg/m2
† Excluding participants who died prior to April 2018

For peer review only

Table 3 shows the multivariable logistic regression for risk factors for outcomes of any hospital admissions, ≥7 hospital admissions and >20 days of hospital stay between 1999 to 2018. Models are presented for all participants, men and women and each risk factor is adjusted for all others for the nine models. Male sex is only included in the models for all participants. As well as age, social class, education and body mass index, the four individual components of Townsend index are modelled. As previously reported, age, male sex, lower education level, manual social class, current cigarette smoking and a body mass index >30 kg/m^2 were all associated with increased hospitalisation. No single component of the Townsend index was more strongly associated for all outcomes for both men and women. However, the unemployment component was most strongly associated with 7 or more hospital admissions. Areas with low car ownership appeared to have a greater association in women than men.

Table 3 | Multivariable logistic regression of risk factors for any hospital admissions, ≥7 hospital admissions and >20 days of hospital stay from 1999 to 2018 in 24977 men and women. Townsend index components using the UK 1991 census

	All subjects OR (95% CI)	p value	Men OR (95% CI)	p value	Women OR (95% CI)	p value
**Outcome of any hospital admissions**						
Male sex	1.13 (1.04–1.23)	0.006				
Age per 10 years	2.08 (1.98–2.20)	< 0.001	2.15 (1.99–2.34)	< 0.001	2.03 (1.89–2.17)	< 0.001
Unemployment Z-Score >0	1.06 (0.92–1.21)	0.426	1.06 (0.87–1.31)	0.560	1.05 (0.88–1.26)	0.604
Households with no car Z-Score >0	1.05 (0.89–1.23)	0.568	0.91 (0.72–1.16)	0.459	1.16 (0.94–1.44)	0.161
Households not owner-occupied Z-Score >0	1.03 (0.90–1.19)	0.625	0.99 (0.81–1.22)	0.948	1.07 (0.89–1.28)	0.479
Household overcrowding Z-Score >0	0.98 (0.88–1.10)	0.760	1.00 (0.84–1.19)	0.983	0.97 (0.84–1.13)	0.717
Manual social class	1.16 (1.06–1.27)	0.001	1.25 (1.08–1.43)	0.002	1.11 (0.98–1.25)	0.106
Low education level	1.35 (1.22–1.50)	< 0.001	1.21 (1.03–1.43)	0.026	1.45 (1.27–1.66)	< 0.001
Current smoker	1.21 (1.06–1.38)	0.006	1.15 (0.94–1.41)	0.174	1.26 (1.05–1.51)	0.013
BMI>30 kg/m^2	1.28 (1.12–1.46)	< 0.001	1.19 (0.97–1.47)	0.095	1.34 (1.13–1.59)	< 0.001
**Outcome of 7 or more hospital admissions**						
Male sex	1.23 (1.16–1.30)	< 0.001				
Age per 10 years	1.49 (1.44–1.54)	< 0.001	1.50 (1.43–1.57)	< 0.001	1.48 (1.42–1.55)	< 0.001
Unemployment Z-Score >0	1.18 (1.08–1.28)	< 0.001	1.17 (1.04–1.33)	0.012	1.18 (1.06–1.33)	0.004
Households with no car Z-Score >0	0.98 (0.88–1.08)	0.641	0.92 (0.79–1.06)	0.253	1.03 (0.90–1.17)	0.692
Households not owner-occupied Z-Score >0	0.93 (0.86–1.02)	0.134	0.91 (0.80–1.04)	0.163	0.95 (0.85–1.08)	0.447
Household overcrowding Z-Score >0	1.08 (1.00–1.16)	0.044	1.07 (0.96–1.19)	0.207	1.08 (0.98–1.20)	0.112
Manual social class	1.15 (1.08–1.22)	< 0.001	1.16 (1.06–1.26)	< 0.001	1.14 (1.06–1.24)	0.001
Low education level	1.14 (1.07–1.21)	< 0.001	1.12 (1.02–1.23)	0.014	1.15 (1.06–1.25)	< 0.001
Current smoker	1.34 (1.23–1.46)	< 0.001	1.25 (1.10–1.41)	< 0.001	1.44 (1.28–1.61)	< 0.001
BMI>30 kg/m^2	1.38 (1.28–1.49)	< 0.001	1.39 (1.24–1.56)	< 0.001	1.38 (1.25–1.52)	< 0.001
**Outcome of more than 20 hospital days**						
Male sex	1.16 (1.10–1.23)	< 0.001				
Age per 10 years	2.68 (2.59–2.78)	< 0.001	2.62 (2.49–2.76)	< 0.001	2.73 (2.61–2.87)	< 0.001
Unemployment Z-Score >0	1.10 (1.01–1.20)	0.034	1.10 (0.96–1.25)	0.161	1.10 (0.98–1.24)	0.111
Households with no car Z-Score >0	1.00 (0.90–1.11)	0.979	0.95 (0.81–1.11)	0.501	1.04 (0.90–1.19)	0.599
Households not owner-occupied Z-Score >0	1.00 (0.92–1.10)	0.916	1.01 (0.88–1.16)	0.849	1.00 (0.88–1.13)	0.958
Household overcrowding Z-Score >0	1.07 (0.99–1.15)	0.100	1.03 (0.92–1.15)	0.622	1.10 (0.99–1.22)	0.071
Manual social class	1.16 (1.09–1.24)	< 0.001	1.19 (1.09–1.31)	< 0.001	1.14 (1.05–1.24)	0.003
Low education level	1.16 (1.09–1.24)	< 0.001	1.18 (1.07–1.30)	< 0.001	1.15 (1.05–1.25)	0.001
Current smoker	1.57 (1.44–1.72)	< 0.001	1.61 (1.41–1.83)	< 0.001	1.54 (1.37–1.74)	< 0.001
BMI>30 kg/m^2	1.61 (1.49–1.74)	< 0.001	1.58 (1.40–1.79)	< 0.001	1.63 (1.48–1.81)	< 0.001

For peer review only

Figure 1 shows graphs of length of hospital stay by quintiles of Townsend index. In the first plot, results are stratified by higher and lower education levels while in the second plot the results are stratified by manual and non-manual social class. Since time in hospital was skewed with some people remaining in hospital for extended periods, those with length of stay longer than 365 days were assigned that value. The graphs demonstrate the disparity between individual socioeconomic factors and hospital stay when area deprivation index is also considered. For example, the mean time spent in hospital for participants with a manual social class differs by only one day for those in quintile 1 of Townsend Index while for those in quintile 5 it differed by five days.

Supplementary table S1 displays logistic regression models for the outcome of more than 20 hospital days for Townsend index in various subgroups. Models are stratified by a dichotomised subgroup: men and women, age above or below 65 years, manual and non-manual social class, lower or higher education level, smoking status, BMI above and below 30 kg/m^2, urban or rural home postcode, and moved house between the year 2000 and 2014. Odds ratios (OR) within all strata were in consistent directions with no interaction by age, smoking status or BMI. However, a significant interaction was observed between social class and level of education and Townsend index ($p_{Interaction}$=0.02 and $p_{Interaction}$=0.02 respectively)

Discussion

Residential area deprivation was associated with future hospital usage independently of individual sociodemographic factors in particular, age, sex, social class and education, as well as lifestyle factors smoking and body mass index in this cohort of middle-aged and older men and women. Study participants in the highest fifth of Townsend Index - those living in the most deprived areas, at or below the national average, were more likely to spend more than 20 days in hospital or be admitted to hospital on more than 7 occasions. There were also significant interactions between residential area deprivation and individual social class and education level. Participants with a manual social class living in an area with higher deprivation index spent longer in hospital than those with manual occupations living in less deprived areas. Similarly, those with lower education level living in more deprived areas had the greatest risk of hospitalisation. This suggests that hospitalisation is greatest when those with poorer individual socioeconomic factors are combined with residential deprivation. We considered a number of possible explanations for these findings.

Study participants were approximately evenly divided into those living in urban and those in rural areas. Moderate deprivation (those with Townsend quintile 2-4) were more likely to live in rural areas while the most deprived (Townsend quintile 5) were predominantly urban dwellers. Study participants did not vary greatly in the distance by road from their home to the Norfolk and Norwich hospital or the time taken for the journey. There is some evidence to suggest that travel time is associated with hospital use [25,26] but there was no strong association in this study.

Strengths and limitations of this study

The EPIC-Norfolk cohort is very well characterised. This enabled us to take into account many potentially confounding variables understood to be related to hospital usage and disease. The UK National Health Service is free at the point of use and consequently income is not a major determinant of hospital admissions. Despite this, social class, education and residential deprivation were all independently related to hospital use.

Townsend Area Deprivation Index is associated with individual sociodemographic factors such as occupational social class and education and other factors including age, sex and BMI. Since all these factors are also related to hospital use, some level of confounding will be present. However, multivariable regression models adjusting for all these variables only modestly attenuated the area deprivation associations. In a Table 3, we stratified by the main confounders and the results remained consistent in the subgroups. The accuracy of the measurement might not be sufficient to ensure adequate adjustment so we cannot exclude the possibility of residual confounding

For peer review only

with known or other unknown factors associated with both Townsend Index and hospital usage. These unknown factors may either attenuate or strengthen the associations. Interactions between area deprivation and individual sociodemographic factors highlighted stronger associations among more deprived groups.

The use of area-based measurements has some limitations. The factors used in the Townsend score may vary in their ability to assess deprivation according to setting. In urban areas, lower car ownership rates may reflect the availability of other transport options and closer proximity of work places and facilities such as shops. In rural areas, overcrowding may be less common while car ownership may be more of a necessity while simultaneously a drain on resources. The deprivation index is based on data from the UK census that only takes place every ten years. Over the period under examination, areas may change becoming more or less deprived while participants may move house to a different area.

Area deprivation was determined by postcode of residence in the year 2000. Study participants who moved house may have been misclassified for some of the period over which hospitalisation was assessed. However, the majority of participants remained in the same location and a sensitivity analysis of non-movers found very similar results to the main analyses. It is not possible to know reasons for such relocation. However, misclassification due to moves or changes over time in residential area deprivation scoring and resultant measurement error would be likely to attenuate any associations with the residential area score.

Differential misclassification in hospital use may be explained by early death rates. Study participants living in more deprived areas may have died earlier and not used hospital services for the full period. However, while the death rate was higher among those living in the most deprived areas, 64.9% of the cohort survived beyond 2018 and models restricted to survivors were more strongly associated with outcome measures than those in the main analysis. Sociodemographic factors may be less relevant for the very seriously ill who require hospital treatment at the end of life.

It may also be possible that individuals did not use NHS facilities but private hospitals differently by socioeconomic status which might explain lower use in the higher sociodemographic groups. However, the use of private hospitals in the Norfolk area over this time period was minimal.

Comparison with other studies

Most studies examining deprivation in the context of health, disease and mortality either rely on area based measures collected for example from census data or from individual level data from questionnaires. We had access to both forms of information, having derived individual social class and education level from self reported questionnaires and area level measures from residential postcode linkage. Hospital based studies using patients as study participants do not have a reliable population based denominator and cannot estimate overall risk in the population. We were able to examine hospital usage over 19 years in a clearly defined community based population using a prospective cohort design. Record linkage of routinely collected hospital episode data gives virtually complete ascertainment as the use of private healthcare in Norfolk over the outcome period was minimal. We have previously reported that age, sex, body mass index and smoking status predict future hospital use in this cohort over a 10 year period of follow-up. In this paper we use national HES data which includes all admissions for hospitals in England and Wales. While 22% of the cohort moved house between the years 2000 and 2014, almost all relocated locally in Norfolk or elsewhere in England and consequently there was virtually no loss to follow-up.

Our results provide further evidence adding to the substantial literature linking deprivation to health. Unlike many studies, we used overall measures of hospital activity, including both elective and emergency admissions and found evidence of an independent association of residential area deprivation not accounted for by known individual factors such as social class and education. Our results also demonstrate that the combination of

For peer review only

residential area deprivation with lower levels of education or manual social class result in the highest levels of hospitalisation. Neither distance from hospital nor urban or rural location explained this finding, since those in the lowest deprivation areas are mainly urban with the shortest travel time to hospital. However, irrespective of travel distance or time, owning or having access to a car would make a considerable difference in being able to access local facilities. Although there may be more regular public transport services in cities, with will vary and cost and limited travel options may restrict access not only to hospital but also to friends and relatives, to better quality supermarkets and to parks and recreational facilities.

Possible explanations of findings

Norfolk is an area of generally low deprivation with >80% of the study population living in areas with deprivations levels below the national average. While levels of deprivation over the study population were sufficient to examine variation in hospital use, very few participants live in areas of high deprivation such as those found in some larger cities in other parts of the country. While it seems likely that those living in more deprived cities or regions would have a socioeconomic gradient in hospital usage more extreme than we were able to observe, our study does not provide any information on the most extreme forms of deprivation.

The Black report concluded that health inequalities were not mainly attributable to failings in the NHS, but rather to many other social inequalities influencing health: income, education, housing, diet, employment, and conditions of work. It suggested two mechanisms for how social determinants influence health: cultural/behavioural and materialist/structuralist. Some authors have pointed out that research on the determinants of health are generally focused on the individual but patterns of population health are unclear without examining structural determinants at the societal level. [27] Townsend's residential deprivation index uses aggregate measures of particular characteristics for people living in an area. It has been used mainly as a surrogate for individual measures of deprivation in many studies. [28] We were not able to examine physical features of the environment in this study. Ecological measurements such as the quality of housing, access to recreational facilities, local services provided, community support and levels of crime may affect health and hospital usage. However, we were able to examine both individual and area level deprivation in the same study participants, and the interaction we observed suggests that there is a higher risk hospitalisation in more deprived areas of residence disproportionately for those with lower individual social class and education. Conversely, individuals with non-manual social class and higher levels of education appear more resilient to hospitalisation irrespective of the level of deprivation of their residence.

Conclusions and policy implications

There is a socioeconomic gradient in hospital usage for factors measured both individually and at area level. Residential area deprivation predicts future hospitalisations, time spent in hospital and number of admissions, independently of individual social class and education level and other behavioural factors. There are significant interactions such that residential area deprivation has greater impact in those with low education level or manual social class. Conversely, higher education level and social class mitigated the association of area deprivation with hospital usage.

For peer review only

References

1 Antonovsky A. Social Class and Illness: A Reconsideration. *Sociological Inquiry* 1967;**37**:311–22. doi:10.1111/j.1475-682X.1967.tb00661.x

2 Black D. Inequalities in Health: Report of a Research Working Group. 1980.

3 Acheson D. *Independent inquiry into inequalities in health: report*. HM Stationery Office 1998.

4 Smith GD, Bartley M, Blane D. The Black report on socioeconomic inequalities in health 10 years on. *BMJ* 1990;**301**:373–7.

5 Health expectancies at birth and at age 65 in the UK, based on 2011 Census health and disability prevalence data - Office for National Statistics. https://www.ons.gov.uk/peoplepopulationandcommunity/healthandsocialcare/healthandlifeexpectancies/bulletins/healthexpectanciesatbirthandatage65intheukbasedon2011censushealthanddisabilityprevalencedata/2010to2012 (accessed 23 May 2018).

6 Life Expectancy at Birth and at Age 65 by Local Areas in England and Wales - Office for National Statistics. https://www.ons.gov.uk/peoplepopulationandcommunity/birthsdeathsandmarriages/lifeexpectancies/bulletins/lifeexpectancyatbirthandatage65bylocalareasinenglandandwales/2015-11-04 (accessed 23 May 2018).

7 Marmot M, Friel S, Bell R, *et al.* Closing the gap in a generation: health equity through action on the social determinants of health. *The Lancet* 2008;**372**:1661–9. doi:10.1016/S0140-6736(08)61690-6

8 Stafford M, Marmot M. Neighbourhood deprivation and health: does it affect us all equally? *Int J Epidemiol* 2003;**32**:357–66. doi:10.1093/ije/dyg084

9 Townsend P, Phillimore P, Beattie A. Health and deprivation. Inequality and the North. *Croom Helm Ltd, London* Published Online First: 1987. doi:10.1016/0168-8510(88)90006-1

10 McFadden E, Luben R, Khaw K-T. Different measures of social class in women and mortality. *Eur J Epidemiol* 2009;**24**:231–6. doi:10.1007/s10654-009-9332-0

11 Asaria M, Doran T, Cookson R. The costs of inequality: whole-population modelling study of lifetime inpatient hospital costs in the English National Health Service by level of neighbourhood deprivation. *J Epidemiol Community Health* 2016;**70**:990–6. doi:10.1136/jech-2016-207447

12 Bernard S, Smith LK. Emergency admissions of older people to hospital: a link with material deprivation. *J Public Health (Oxf)* 1998;**20**:97–101. doi:10.1093/oxfordjournals.pubmed.a024727

13 Conway R, Galvin S, Coveney S, *et al.* Deprivation as an outcome determinant in emergency medical admissions. *QJM* 2013;**106**:245–51. doi:10.1093/qjmed/hcs233

14 Conway R, Byrne D, O'Riordan D, *et al.* Deprivation index and dependency ratio are key determinants of emergency medical admission rates. *European Journal of Internal Medicine* 2015;**26**:709–13. doi:10.1016/j.ejim.2015.09.010

15 Moore L, Cisse B, Batomen Kuimi BL, *et al.* Impact of socio-economic status on hospital length of stay following injury: a multicenter cohort study. *BMC Health Serv Res* 2015;**15**. doi:10.1186/s12913-015-0949-2

16 Macintyre S, Maciver S, Sooman A. Area, Class and Health: Should we be Focusing on Places or People? *Journal of Social Policy* 1993;**22**:213. doi:10.1017/S0047279400019310

17 Smith GD, Hart C, Watt G, *et al.* Individual social class, area-based deprivation, cardiovascular disease risk factors, and mortality: the Renfrew and Paisley Study. *J Epidemiol Community Health* 1998;**52**:399–405.

For peer review only

18 Shohaimi S, Luben R, Wareham N, *et al.* Residential area deprivation predicts smoking habit independently of individual educational level and occupational social class. A cross sectional study in the Norfolk cohort of the European Investigation into Cancer (EPIC-Norfolk). *Journal of Epidemiology & Community Health* 2003;**57**:270–6. doi:10.1136/jech.57.4.270

19 Luben R, Hayat S, Wareham N, *et al.* Predicting admissions and time spent in hospital over a decade in a population-based record linkage study: the EPIC-Norfolk cohort. *BMJ Open* 2016;**6**:e009461. doi:10.1136/bmjopen-2015-009461

20 Hayat SA, Luben R, Keevil VL, *et al.* Cohort Profile: A prospective cohort study of objective physical and cognitive capability and visual health in an ageing population of men and women in Norfolk (EPIC-Norfolk 3). *Int J Epidemiol* 2014;**43**:1063–72. doi:10.1093/ije/dyt086

21 Day N, Oakes S, Luben R, *et al.* EPIC-Norfolk: study design and characteristics of the cohort. European Prospective Investigation of Cancer. *Br J Cancer* 1999;**80 Suppl 1**:95–103.

22 Bland R. Measuring 'Social Class': A Discussion of the Registrar-General's Classification. *Sociology* 1979;**13**:283–91. doi:10.1177/003803857901300209

23 Luxen D, Vetter C. Real-time routing with OpenStreetMap data. In: *Proceedings of the 19th ACM SIGSPATIAL International Conference on Advances in Geographic Information Systems*. Chicago, Illinois: : ACM 2011. 513–6. doi:10.1145/2093973.2094062

24 Boyle S. United Kingdom (England): Health system review. *Health Syst Transit* 2011;**13**:1–483, xix–xx.

25 Kelly C, Hulme C, Farragher T, *et al.* Are differences in travel time or distance to healthcare for adults in global north countries associated with an impact on health outcomes? A systematic review. *BMJ Open* 2016;**6**:e013059. doi:10.1136/bmjopen-2016-013059

26 Nicholl J, West J, Goodacre S, *et al.* The relationship between distance to hospital and patient mortality in emergencies: an observational study. *Emerg Med J* 2007;**24**:665–8. doi:10.1136/emj.2007.047654

27 Lynch JW, Kaplan GA. Understanding How Inequality in the Distribution of Income Affects Health. *J Health Psychol* 1997;**2**:297–314. doi:10.1177/135910539700200303

28 Macintyre S, Maciver S, Sooman A. Area, Class and Health: Should we be Focusing on Places or People? *Journal of Social Policy* 1993;**22**:213–34. doi:10.1017/S0047279400019310

For peer review only

Supporting information

Supplementary Table S1 | Multivariable logistic regression of Townsend Index and more than twenty hospital days in subgroups

Figure 1: Hospitalisation by deprivation index. Length of hospital stay over 19 years of follow-up by quintiles of Townsend Index grouped by categories of education level and categories of social class. Low education level is defined as those having no qualifications and high education as those with at least some qualifications at secondary level or above. Length of stay is truncated to 365 days for those staying longer than 365 days. Interaction tested using multivariable adjusted linear regression with covariables age, sex, education level (higher/lower), body mass index (≤30/>30) smoking status (current/non-current)

For peer review only

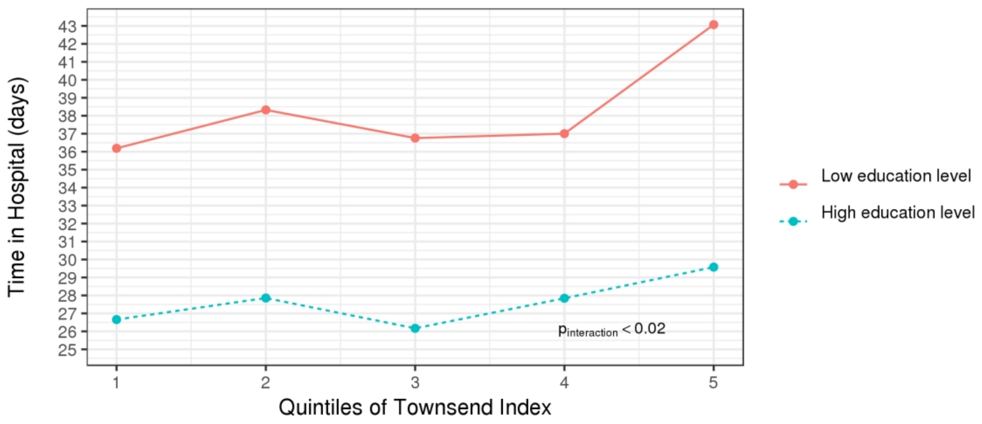
Time in Hospital (days)
43
42
41
40
39
38
37
36
35
34
33
32
31
30
29
28
27
26
25
1
2
3
4
5
Quintiles of Townsend Index
$p_{interaction} < 0.02$
Low education level
High education level

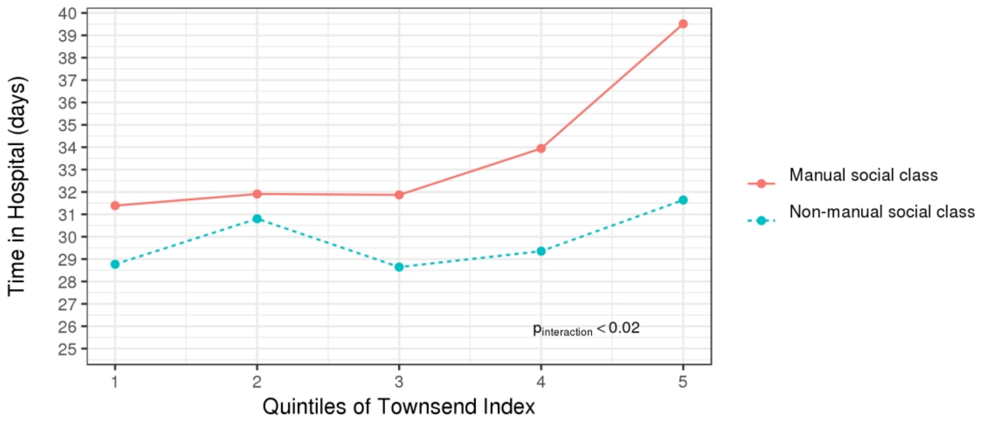
Time in Hospital (days)
40
39
38
37
36
35
34
33
32
31
30
29
28
27
26
25
1
2
3
4
5
Quintiles of Townsend Index
$p_{interaction} < 0.02$
Manual social class
Non-manual social class

Supporting information

Supplementary Table S1 | Multivariable logistic regression of Townsend Index and more than twenty hospital days in subgroups

	Townsend Index OR (95% CI)†
**Men and women**	
Men (n=11214)	1.01 (0.99–1.03)
Women (n=13763)	1.03 (1.01–1.05)
**By age above and below 65 years**	
Less than 65 years (n=17343)	1.03 (1.01–1.05)
65 years and above (n=7634)	1.01 (0.98–1.03)
**Manual and non-manual social class**	
Non-manual (n=14691)	1.02 (1.00–1.04)
Manual (n=9741)	1.03 (1.00–1.05)
**By level of education**	
Higher level (n=15841)	1.01 (0.99–1.03)
Lower level (n=9118)	1.03 (1.01–1.06)
**By smoking status**	
Former or never smoker (n=21864)	1.02 (1.01–1.04)
Current smoker (n=2895)	1.00 (0.97–1.04)
**By level of body mass index**	
BMI ≤ 30 kg/m² (n=21124)	1.02 (1.01–1.04)
BMI > 30 kg/m² (n=3800)	1.01 (0.98–1.04)
**Urban or rural home postcode**	
Urban (n=11214)	1.01 (1.00–1.03)
Rural (n=13763)	1.03 (1.01–1.06)
**Moved house between 2000 and 2014**	
Moved house (n=5355)	1.04 (1.01–1.07)
Did not move house (n=18728)	1.01 (1.00–1.03)

Logistic regression per unit Townsend Index. † Adjusted for age, current smoking, BMI (categories)

For peer review only

For peer review only - http://bmjopen.bmj.com/site/about/guidelines.xhtml

STROBE Statement—Checklist of items that should be included in reports of ***cohort studies***

	Item No	Recommendation	Page No
**Title and abstract**	1	(*a*) Indicate the study's design with a commonly used term in the title or the abstract	1
		(*b*) Provide in the abstract an informative and balanced summary of what was done and what was found	2
**Introduction**			
Background/rationale	2	Explain the scientific background and rationale for the investigation being reported	4
Objectives	3	State specific objectives, including any prespecified hypotheses	4
**Methods**			
Study design	4	Present key elements of study design early in the paper	5 and title
Setting	5	Describe the setting, locations, and relevant dates, including periods of recruitment, exposure, follow-up, and data collection	5-6
Participants	6	(*a*) Give the eligibility criteria, and the sources and methods of selection of participants. Describe methods of follow-up	6-7
		(*b*) For matched studies, give matching criteria and number of exposed and unexposed	
Variables	7	Clearly define all outcomes, exposures, predictors, potential confounders, and effect modifiers. Give diagnostic criteria, if applicable	7
Data sources/ measurement	8*	For each variable of interest, give sources of data and details of methods of assessment (measurement). Describe comparability of assessment methods if there is more than one group	6-7
Bias	9	Describe any efforts to address potential sources of bias	11-12
Study size	10	Explain how the study size was arrived at	7
Quantitative variables	11	Explain how quantitative variables were handled in the analyses. If applicable, describe which groupings were chosen and why	7
Statistical methods	12	(*a*) Describe all statistical methods, including those used to control for confounding	9-11
		(*b*) Describe any methods used to examine subgroups and interactions	Table S1

		(*c*) Explain how missing data were addressed	7
		(*d*) If applicable, explain how loss to follow-up was addressed	12
		(*e*) Describe any sensitivity analyses	S1, Covariables in subgroups
**Results**			
Participants	13*	(a) Report numbers of individuals at each stage of study—eg numbers potentially eligible, examined for eligibility, confirmed eligible, included in the study, completing follow-up, and analysed	5, 7
		(b) Give reasons for non-participation at each stage	7
		(c) Consider use of a flow diagram	
Descriptive data	14*	(a) Give characteristics of study participants (eg demographic, clinical, social) and information on exposures and potential confounders	Table 1
		(b) Indicate number of participants with missing data for each variable of interest	6
		(c) Summarise follow-up time (eg, average and total amount)	Stated in title
Outcome data	15*	Report numbers of outcome events or summary measures over time	Table 1

For peer review only - http://bmjopen.bmj.com/site/about/guidelines.xhtml

Main results	16	(*a*) Give unadjusted estimates and, if applicable, confounder-adjusted estimates and their precision (eg, 95% confidence interval). Make clear which confounders were adjusted for and why they were included	8 (Table 2)
		(*b*) Report category boundaries when continuous variables were categorized	6 (Statistical analysis)
		(*c*) If relevant, consider translating estimates of relative risk into absolute risk for a meaningful time period	
Other analyses	17	Report other analyses done—eg analyses of subgroups and interactions, and sensitivity analyses	S1, Covariables in subgroups
**Discussion**			
Key results	18	Summarise key results with reference to study objectives	10
Limitations	19	Discuss limitations of the study, taking into account sources of potential bias or imprecision. Discuss both direction and magnitude of any potential bias	10-11
Interpretation	20	Give a cautious overall interpretation of results considering objectives, limitations, multiplicity of analyses, results from similar studies, and other relevant evidence	12
Generalisability	21	Discuss the generalisability (external validity) of the study results	
**Other information**			
Funding	22	Give the source of funding and the role of the funders for the present study and, if applicable, for the original study on which the present article is based	

*Give information separately for exposed and unexposed groups.

Note: An Explanation and Elaboration article discusses each checklist item and gives methodological background and published examples of transparent reporting. The STROBE checklist is best used in conjunction with this article (freely available on the Web sites of PLoS Medicine at http://www.plosmedicine.org/, Annals of Internal Medicine at http://www.annals.org/, and Epidemiology at http://www.epidem.com/). Information on the STROBE Initiative is available at http://www.strobe-statement.org.

For peer review only - http://bmjopen.bmj.com/site/about/guidelines.xhtml

Residential area deprivation and risk of subsequent hospital admission in a British population: the EPIC-Norfolk cohort

Journal:	*BMJ Open*
Manuscript ID	bmjopen-2019-031251.R1
Article Type:	Research
Date Submitted by the Author:	18-Oct-2019
Complete List of Authors:	Luben, Robert; University of Cambridge, Department of Public Health and Primary Care<br>Hayat, Shabina; University of Cambridge, Department of Public Health & Primary Care<br>Khawaja, Anthony; Moorfields Eye Hospital, NIHR Biomedical Research Centre<br>Wareham, Nicholas; University of Cambridge School of Clinical Medicine, MRC Epidemiology Unit<br>Pharoah, Paul; University of Cambridge, Department of Public Health and Primary Care<br>Khaw, Kay-Tee; University of Cambridge, Public Health and Primary Care
**Primary Subject Heading**:	Epidemiology
Secondary Subject Heading:	Health services research, Health policy
Keywords:	EPIDEMIOLOGY, Health policy < HEALTH SERVICES ADMINISTRATION & MANAGEMENT, PUBLIC HEALTH, SOCIAL MEDICINE

SCHOLARONE™
Manuscripts

Residential area deprivation and risk of subsequent hospital admission in a British population: the EPIC-Norfolk cohort

Robert Luben[1], Shabina Hayat[1], Anthony Khawaja[2], Nicholas Wareham[3], Paul Pharoah[1] , Kay-Tee Khaw[1]

1 Department of Public Health and Primary Care, Institute of Public Health, University of Cambridge, Cambridge, United Kingdom
2 NIHR Biomedical Research Centre, Moorfields Eye Hospital NHS Foundation Trust and UCL Institute of Ophthalmology, London, UK
3 MRC Epidemiology Unit, University of Cambridge School of Clinical Medicine, Cambridge United Kingdom
Correspondence to: Robert Luben, Department of Public Health and Primary Care, Strangeways Research Laboratory, Worts Causeway, Cambridge, UK. Email: robert.luben@phpc.cam.ac.uk Telephone: +44 (0)1223 748691

For peer review only

Word count: 4432

October 17, 2019

ABSTRACT

OBJECTIVES

To investigate whether residential area deprivation index predicts subsequent admissions to hospital and time spent in hospital independently of individual social class and lifestyle factors.

DESIGN

Prospective population based study.

SETTING

The EPIC-Norfolk Study.

PARTICIPANTS

11 214 men and 13 763 women in the general population, aged 40-79 years at recruitment (1993 to 1997), alive in 1999.

MAIN OUTCOME MEASURE

Total admissions to hospital and time spent in hospital during a 19 year time period (1999 to 2018).

RESULTS

Compared to those with residential Townsend Area Deprivation Index lower than the average for England and Wales, those with a higher than average deprivation index had a higher likelihood of spending more than twenty days in hospital multivariable adjusted odds ratio (OR) 1.18 (95% confidence interval (CI) 1.07 to 1.29) and having 7 or more admissions OR 1.11 (95% CI 1.02 to 1.22) after adjustment for age, sex, smoking status, education, social class, and body mass index. Occupational social class and educational attainment modified the association between area deprivation and hospitalisation; those with manual social class and lower education level were at greater risk of hospitalisation when living in an area with higher deprivation index ($p_{\text{Interaction}}$=0.025 and $p_{\text{Interaction}}$=0.020 respectively), while the risk for non-manual and more highly educated participants did not vary greatly by area of residence.

CONCLUSION

Residential area deprivation predicts future hospitalisations, time spent in hospital and number of admissions, independently of individual social class and education level and other behavioural factors. There are significant interactions such that residential area deprivation has greater impact in those with low education level or manual social class. Conversely, higher education level and social class mitigated the association of area deprivation with hospital usage.

For peer review only

STRENGTHS AND LIMITATIONS OF THIS STUDY

- This study is able to examine hospital activity using a prospective cohort design in a population of community-dwelling participants with clearly defined population denominators.
- It uses a large cohort of middle aged and older men and women with 19 years of follow-up time and detailed measurements of demographic and behavioural indicators.
- Both area based census measures and individual social class and education level from questionnaires are used.
- Differential misclassification in hospital use may be explained by early death rates.
- Socioeconomic determinants of hospitalisation were examined in the context of UK National Health Service hospitals which are free at the point of use and so not directly influenced by income.

Acknowledgements: The authors would like to thank the participants, general practitioners, and staff of EPIC-Norfolk.

Contributors: KTK,NW SH and RL were involved in the conception and design of the study. RL drafted the manuscript, with support from KTK and PP. AK contributed to data interpretation. RL was responsible for external data linkage. SH and RL contributed to data collection and acquisition. All authors read and critically revised the manuscript and approved the final manuscript. RL is the guarantor. The corresponding author attests that all listed authors meet authorship criteria and that no others meeting the criteria have been omitted.

Funding: EPIC-Norfolk infrastructure and core functions are supported by grants from the Medical Research Council (G0401527) and Cancer Research UK (C864/A8257).

Competing interests: All authors have completed the ICMJE uniform disclosure form at www.icmje.org/coi_disclosure.pdf and report: RL,SH,KTK and NW report grants from MRC and CRUK during the conduct of the study; no support from any organisation for the submitted work; no financial relationships with any organisations that might have an interest in the submitted work in the previous three years; no other relationships or activities that could appear to have influenced the submitted work.

Ethical approval: The work was approval by the East Norfolk and Waveney NHS research governance committee (2005EC07L) and the Norfolk research ethics committee (05/Q0101/191). All participants gave informed signed consent for study participation including access to medical records.

Data sharing: The authors will make the dataset available under a Data Transfer Agreement to any bona fide researcher who wishes to obtain the dataset in order to undertake a replication analysis. Although the dataset is anonymised, the breadth of the data included and the multiplicity of variables that are included in this analysis file as primary variables or confounding factors, means that provision of the dataset to other researchers without a Data Transfer Agreement would constitute a risk.

Transparency: The lead author (the manuscript's guarantor) affirms that the manuscript is an honest, accurate, and transparent account of the study being reported; that no important aspects of the study have been omitted; and that any discrepancies from the study as planned (and, if relevant, registered) have been explained.

Open access: This is an Open Access article distributed in accordance with the terms of the Creative Commons Attribution (CC BY 4.0) license, which permits others to distribute, remix, adapt and build upon this work, for commercial use, provided the original work is properly cited. See: http://creativecommons.org/licenses/by/4.0/

Patient and public involvement: The EPIC-Norfolk Study have an active Participants Advisory Panel which meets quarterly to advise on research protocols, suggest ideas and provide feedback on the research including proposed new studies and collaborations. All participants of the EPIC-Norfolk study are informed about the study through regular newsletters as well as public meetings. Information is also disseminated through local community talks in the Norfolk area and science festivals.

Exclusive licence: The Corresponding Author has the right to grant on behalf of all authors and does grant on behalf of all authors, a worldwide licence to the Publishers and its licensees in perpetuity, in all forms, formats and media (whether known now or created in the future), to i) publish, reproduce, distribute, display and store the Contribution, ii) translate the Contribution into other languages, create adaptations, reprints, include within collections and create summaries, extracts and/or, abstracts of the Contribution, iii) create any other derivative work(s) based on the Contribution, iv) to exploit all subsidiary rights in the Contribution, v) the inclusion of electronic links from the Contribution to third party material where-ever it may be located; and, vi) licence any third party to do any or all of the above.

The Corresponding Author has the right to grant on behalf of all authors and does grant on behalf of all authors, an exclusive licence (or non exclusive for government employees) on a worldwide basis to the BMJ Publishing Group Ltd to permit this article (if accepted) to be published in BMJ editions and any other BMJPGL products and sublicences such use and exploit all subsidiary rights, as set out in our licence.

For peer review only

Introduction

The considerable differences in mortality by social class are well documented [1–4] with those in higher social classes having a typical life expectancy several years longer than those with the lowest. Similarly, life expectancy and health expectancy varies between UK cities and regions with large variations in expected years of life in good health. [5,6] Despite increasing overall life expectancy, inequality remains with lower life and health expectancy observed more often in disadvantaged groups. While lifestyle factors may account for some part of this, the reported differences in death rates cannot be explained by individual behaviour alone. [7,8] Material deprivation was defined by Townsend as 'a state of observable and demonstrable disadvantage relative to the local community or wider society … to which an individual, family or group belongs'. Deprivation indices use factors such as unemployment, the standard of housing, overcrowding and rates of car ownership which together can assess the level of deprivation within a neighbourhood. [9]

Hospitalisation can be measured using the frequency of admission or the length of stay. When measured over a period of time, the outcome represents burden of resources that might be attributable to a population. Inequality in healthcare utilisation favouring patients who are better off is apparent in half of the OECD countries. [10–12] The UK National Health Service is free at the point of use and consequently should provide equitable healthcare not constrained by ability to pay.

Socioeconomic determinants of hospitalisation have been examined using individual level exposures such as occupational social class, income and education and at area level using various deprivation indices but few studies have both individual and area based measures. Individual occupational social class, income and level of education have all been reported to be associated with chronic disease risk. [13,14] We previously reported that a range of simple demographic and behavioural indicators are related to the future probability of cumulative hospital admissions and bed days. [15] Increasing age and male sex and the modifiable factors current cigarette smoking, body mass index (BMI) >30 kg/m^2, manual social class and low education level were all associated with higher future hospital usage over a ten year period. Area based deprivation measures, available routinely in the UK using postal code linkage, have also reported associations with hospital usage. [16–20] However, the participants in such studies are often limited to those attending hospital and so a suitable population denominator is lacking. Studies reporting health associations for both individual and area measures are less common [21–23] and we are unaware of any studies examining the independent association of residential area deprivation on subsequent hospital usage.

In this paper we examine residential area deprivation using the Townsend deprivation index with subsequent hospital usage over a 19 year period. We explore the independent contribution of residential area deprivation in men and women participants of the EPIC-Norfolk study and its association with future hospitalisation after allowing for the individual level factors previously shown to be associated. We also examine possible interactions between area and individual deprivation measures. Our aim is to determine whether factors such as material living conditions, poor quality housing and poor infrastructure are associated with subsequent hospitalisation in a setting where access to healthcare is unconstrained by ability to pay.

Methods

We used data collected as part of the European Prospective Investigation into Cancer in Norfolk (EPIC-Norfolk), a general population cohort.

For peer review only - http://bmjopen.bmj.com/site/about/guidelines.xhtml

Study design

EPIC-Norfolk is a cohort of men and women living in Norfolk. Recruitment took place between 1993 and 1997 at 35 general practices with invitations sent to all those registered with the practices aged 40–79 years. The design and recruitment of the study has been previously described in detail. [24,25] Briefly, a total of 77 630 invitations were sent to adults registered at participating GP practices; 30 445 (40%) consented to participate in the study of whom 25 639 men and women completed a lifestyle questionnaire and attended a health examination. The study has ethics committee approval from Norfolk Research Ethics Committee (Rec Ref: 98CN01) and all participants gave informed signed consent for study participation including access to medical records. Residential postcode, recorded at the end of recruitment, was used to link to the UK 1991 national census data. [26] Between 1999 and 2018 the cohort was linked to databases maintained by the East Norfolk Primary Health Care Trust and later to national databases held by NHS Digital. [27] Hospital Episode Statistics (HES) records which included admission and discharge dates were used to calculate time in hospital and numbers of admissions. Contiguous admissions were merged and counted as a single admission. Details of linkage of the EPIC-Norfolk cohort participants to hospital records have been previously reported. [15]

Residential area deprivation score for participants

The Townsend Index is an area deprivation measurement calculated using four components: the percentage unemployed of economically active residents over 16 years old, the percentage of households with no car, the percentage of households not owner occupied, and the percentage of households with more than one person per room. These are respectively: a measure of lack of material resources and insecurity, a proxy for current income, a proxy for current wealth, and a measure of material living conditions. The index used in this study was constructed using data collected at the 1991 UK census, which takes place every ten years. Each Townsend component was calculated at Enumeration District (ED), a small area containing an average 175 households used by the census administrators both as output areas and for data collection. Townsend components were then standardised as z-scores at ED level for England and Wales. Study participants were linked to an ED using their home postcode at the end of recruitment in the year 2000. The link was then used to establish a residential Townsend Index for each individual.

Covariables

Participants' height and weight were measured in light clothing without shoes by trained nurses in a clinic setting as part of a health examination between 1993 and 1997. Height was measured to the nearest 0.1cm using a stadiometer (Chasemores, UK) and weight to the nearest 0.1kg. BMI was calculated using measured weight in kilograms divided by measured height^2 in square metres.

Participants completed a lifestyle questionnaire which included questions about their and their partner's current and past employment. Occupational social class was defined according to the Registrar General's classification [28] and dichotomised into non-manual and manual social classes. Professional, managerial and technical and non-manual skilled occupations (codes I, II and IIIa respectively) were classed as non-manual while manual skilled, partly skilled and unskilled (codes IIIb, IV and V respectively) were classed as manual. Social class for men used (in order of priority) their own current employment, own past employment, partner's current employment or partner's past employment according to whether a social class classification could be defined for a given occupation type. Similarly, social class for women used (in order of priority) their partner's current employment, partner's past employment, own current employment, own past employment. The use of partner's social class for women in the EPIC-Norfolk cohort born between 1918 and 1948 has been previously discussed. [29]

The question "Do you have any of the following qualifications" together with a list of common UK qualifications was used to establish educational attainment. Participants were categorised according to the highest qualification they attained: those with no formal qualifications, those with formal qualifications usually associated with

For peer review only - http://bmjopen.bmj.com/site/about/guidelines.xhtml

completing school aged between 16 and 18 years, and those with degree level qualifications.

Smoking status was derived from two questions: “Have you ever smoked as much as one cigarette a day for as long as a year” and “Do you smoke cigarettes now”. The responses to both questions were “yes” or “no” and participants were asked to leave the second question blank if they answered “no” to the first.

Travel time and travel distance between participants home postcode and the Norfolk and Norwich University Hospitals NHS Foundation Trust was calculated using the Open Source Routing Machine (OSRM) [30] which calculates the shortest path between two points over the road network. Postcode of home residence was used to establish if a participant had moved house over the follow-up period. It was available at two points in time: in the year 2000 and the year 2014. Participants whose postcode or house location changed during the period were classified as having moving house but were not excluded from the analyses. Urban and rural categories were established using the 1991 census.

Ascertainment of hospital usage and mortality through record linkage

Details of linkage of the EPIC-Norfolk cohort participants to hospital records have been previously reported. [15] Briefly, linkage using unique NHS numbers was performed between 1999 and 2018 to databases maintained by the East Norfolk Primary Health Care Trust and to national databases held by NHS Digital. [27] All hospital activity for EPIC-Norfolk participants was captured wherever they were treated in England and Wales. Hospital episode statistics (HES) records which included admission and discharge dates were used to calculate time in hospital and numbers of admissions. Contiguous admissions were merged and counted as a single admission.

Statistical analysis

For the current analyses, we excluded the 625 men and women from the baseline cohort who died before 1999. A further 37 who did not have a valid UK postcode were excluded leaving 24 977 participants. Dichotomous variables were created for the three socioeconomic status variables. Occupational social class was categorised into non-manual and manual: social classes I, II, and III non-manual were classified as ‘non-manual’, while social classes III manual, IV, and V were classified as ‘manual’. Educational level was categorised into ‘Higher level’ (which includes those with qualifications at secondary level or above) and ‘Lower level’ (those with no qualification). Townsend deprivation index was divided into quintiles. Lower Townsend scores correspond to lower levels of deprivation. Quintiles 1-4 are all below zero and hence below (less deprived than) the national average for England and Wales. Quintile 5 (-0.64, 6.99] corresponds to Townsend scores close to or above the national average (more deprived). Overall Townsend score and components were also dichotomised with scores below zero defined as ‘less deprived’ and scores above 0 as ‘more deprived’. Hospital admissions were categorised into five groups: 0, 1, 2–3, 4–6, and ≥7 while time in hospital was divided into categories: None, Day case, 2–5 days, 6–20 days, and >20 days. The cut-points were chosen to be consistent with earlier work. [15] Since time in hospital was skewed with some people remaining in hospital for extended periods, length of stay longer than 365 days was truncated for graphical presentation. A dichotomous urban/rural variable was defined with “urban” and “urban sparse” as urban and “town”, “village” or “hamlet” as rural. Three dichotomous outcome categories were calculated: any hospital admissions (vs no admissions), 7 or more admissions (vs fewer than 7) using total admissions, and greater than 20 hospital days (vs 20 or fewer) using total bed days (overnight stays) and day cases. Multivariable logistic regression was used for all models. All analyses were performed using the R statistical language (R Foundation for Statistical Computing, Vienna, Austria version 3.5.3 with packages knitr, Gmisc, ggplot2, tidyverse, intubate)

Results

Table 1 shows descriptive characteristics by quintiles of residential Townsend Index for 11 214 men and 13 763 women. The majority (n=20 996) of study participants had deprivation index below zero while n=3 981,

For peer review only

approximately corresponding to those in quintile 5, had levels above the national average. Participants in quintile 5 were much more likely to live in an urban setting (70.2%) while those in quintiles 2,3 and 4 were more likely to live in a rural location. Travel distance was lowest for participants in quintile 1 and 5, perhaps due to a higher proportion living in cities and travel times followed a similar pattern. Participants in quintile 5 were the most likely to move house (26.1% between 2000 and 2014). Hospital admissions and time in hospital are shown for both the full cohort and restricted to those who attended hospital. 10.5% of study participants had no admissions over the 19 years from 1999 to 2018.

Table 1 | Descriptive characteristics by quintiles of Townsend Index

	Total	Quintile 1 [-6.74,-3.81]	Quintile 2 (-3.81,-2.94]	Quintile 3 (-2.94,-2.09]	Quintile 4 (-2.09,-0.64]	Quintile 5 (-0.64, 6.99]	P-value
**Sex [n (%)]**							
Men	11214 (44.9)	2271 (45.2)	2262 (45.4)	2280 (45.2)	2226 (45.0)	2175 (43.7)	0.41
Women	13763 (55.1)	2752 (54.8)	2723 (54.6)	2760 (54.8)	2722 (55.0)	2806 (56.3)	
**Age, years**							
Mean ±SD	59.0 ±9.3	58.8 ±9.0	59.0 ±9.2	58.8 ±9.2	59.2 ±9.4	59.4 ±9.5	0.002
**Body mass index, kg/m²**							
Mean ±SD	26.4 ±3.9	26.1 ±3.8	26.3 ±3.8	26.4 ±3.9	26.5 ±4.0	26.5 ±4.1	< 0.001
**Cigarette smoking [n (%)]**							
Current	2895 (11.7)	457 (9.2)	501 (10.1)	569 (11.4)	575 (11.7)	793 (16.1)	< 0.001
Former	10411 (42.0)	2033 (40.7)	2083 (42.1)	2044 (41.0)	2132 (43.4)	2119 (43.1)	
Never	11453 (46.3)	2502 (50.1)	2361 (47.7)	2378 (47.6)	2203 (44.9)	2009 (40.8)	
**Social class dichotomised [n (%)]**							
Non-manual	14691 (60.1)	3336 (67.4)	3170 (64.8)	2950 (59.8)	2840 (58.9)	2395 (49.5)	< 0.001
Manual	9741 (39.9)	1610 (32.6)	1722 (35.2)	1985 (40.2)	1982 (41.1)	2442 (50.5)	
**Level of education [n (%)]**							
Higher level	15841 (63.5)	3439 (68.5)	3373 (67.7)	3218 (63.9)	3084 (62.4)	2727 (54.8)	< 0.001
Lower level	9118 (36.5)	1584 (31.5)	1611 (32.3)	1819 (36.1)	1858 (37.6)	2246 (45.2)	
**Travel distance to hospital, km**							
Mean ±SD	20.4 ±13.1	16.5 ±11.3	20.6 ±12.1	22.0 ±12.2	25.2 ±13.2	17.5 ±14.5	< 0.001
**Travel time to hospital, minutes**							
Mean ±SD	20.8 ±10.3	18.0 ±8.9	20.8 ±9.5	21.9 ±9.4	24.4 ±10.6	19.0 ±11.6	< 0.001
**Urban or rural location [n (%)]**							
Urban	11214 (44.9)	2500 (49.8)	1832 (36.8)	1810 (35.9)	1575 (31.8)	3497 (70.2)	< 0.001
Rural	13763 (55.1)	2523 (50.2)	3153 (63.2)	3230 (64.1)	3373 (68.2)	1484 (29.8)	
**Moved house between 2000 and 2014 [n (%)]**							
Moved house	5355 (22.2)	963 (19.8)	972 (20.4)	1091 (22.4)	1060 (22.4)	1269 (26.1)	< 0.001
Did not move house	18728 (77.8)	3903 (80.2)	3799 (79.6)	3774 (77.6)	3662 (77.6)	3590 (73.9)	
**Deaths prior to March 2018 [n (%)]**							
Dead	8727 (35.0)	1630 (32.5)	1704 (34.3)	1703 (33.9)	1781 (36.1)	1909 (38.4)	< 0.001
Alive	16198 (65.0)	3386 (67.5)	3270 (65.7)	3327 (66.1)	3155 (63.9)	3060 (61.6)	
**Hospital activity 1999-2018**							
No admissions	2628 (10.5)	543 (10.8)	528 (10.6)	539 (10.7)	559 (11.3)	459 (9.2)	0.011
One or more admissions	22316 (89.5)	4476 (89.2)	4449 (89.4)	4494 (89.3)	4383 (88.7)	4514 (90.8)	
7 or more admissions	16497 (66.1)	3417 (68.1)	3295 (66.2)	3332 (66.2)	3291 (66.6)	3162 (63.6)	< 0.001
>20 hospital days	15144 (60.7)	3185 (63.5)	3054 (61.4)	3097 (61.5)	2959 (59.9)	2849 (57.3)	< 0.001
**Time spent in hospital 1999-2018, days**							
Full cohort 1999-2018, mean ±SD	32.8 ±63.0	30.5 ±54.9	33.0 ±69.0	31.2 ±57.2	32.5 ±62.9	37.0 ±69.6	< 0.001
Hospital attenders 1999-2018, mean ±SD	36.7 ±65.6	34.2 ±57.0	36.9 ±72.0	34.9 ±59.5	36.6 ±65.7	40.8 ±72.0	< 0.001
**Number of inpatient admissions 1999-2018**							
Full cohort 1999-2018, mean ±SD	7.5 ±26.0	7.3 ±22.8	8.2 ±36.1	7.6 ±30.8	6.7 ±11.7	7.6 ±21.4	0.073
Hospital attenders 1999-2018, mean ±SD	8.4 ±27.3	8.2 ±24.0	9.2 ±38.1	8.6 ±32.5	7.6 ±12.1	8.4 ±22.3	0.095

Round brackets in intervals denote strict inequalities; square brackets denote non-strict inequalities

For peer review only

Table 2 shows the multivariable logistic regression for quintiles of Townsend Index and three outcomes: any hospital admissions, ≥7 hospital admissions, and >20 days of hospital stay between 1999 to 2018. Model 1 is adjusted for age and sex while model 2 is additionally adjusted for manual social class, lower education level, current cigarette smoking and body mass index >30 kg/m2. Additionally, each model is repeated in the subset of participants who survived to the end of the follow-up period. Compared to those with Townsend Deprivation Index quintiles 1 to 4 (lower than the average for England and Wales), those with a deprivation index in quintile 5 had a higher risk of spending more than 20 days in hospital multivariable adjusted odds ratio (OR) 1.18 (95% confidence interval (CI) 1.07–1.29) and for 7 or more hospital admissions OR 1.11 (95% CI 1.02–1.22), but there was no association for any hospital admission. The multivariable adjusted p-value for trend across quintiles of Townsend Index was 0.001 for more than 20 hospital days and 0.107 for 7 or more admissions. Associations in the subset of participants surviving to March 2018 (n=16 198) were higher than those for the full cohort.

Table 2 | Multivariable logistic regression of risk factors by quintiles of Townsend Index for any hospital admissions, ≥7 hospital admissions and >20 days of hospital stay from 1999 to 2018 in 24 977 men and women and in a subset of 16 198 men and women alive in March 2018)

	Quintile 1 [-6.74,-3.81]	Quintile 2 (-3.81,-2.94]	Quintile 3 (-2.94,-2.09]	Quintile 4 (-2.09,-0.64]	Quintile 5 (-0.64, 6.99]	p (trend)
**Outcome of any hospital admissions**						
Model 1	1.00	1.02 (0.89–1.16)	1.01 (0.89–1.15)	0.93 (0.82–1.05)	1.17 (1.02–1.34)	0.175
Model 1†	1.00	1.05 (0.91–1.21)	1.03 (0.89–1.18)	0.95 (0.83–1.09)	1.25 (1.08–1.45)	0.056
Model 2	1.00	1.00 (0.88–1.14)	1.00 (0.87–1.14)	0.90 (0.79–1.03)	1.09 (0.95–1.26)	0.731
Model 2†	1.00	1.04 (0.90–1.20)	1.01 (0.88–1.16)	0.91 (0.79–1.05)	1.18 (1.01–1.37)	0.341
**Outcome of 7 or more hospital admissions**						
Model 1	1.00	1.08 (0.99–1.18)	1.09 (1.00–1.18)	1.05 (0.97–1.15)	1.20 (1.10–1.31)	< 0.001
Model 1†	1.00	1.06 (0.95–1.19)	1.15 (1.03–1.29)	1.15 (1.03–1.29)	1.39 (1.25–1.56)	< 0.001
Model 2	1.00	1.07 (0.98–1.16)	1.05 (0.96–1.15)	1.01 (0.92–1.10)	1.11 (1.02–1.22)	0.107
Model 2†	1.00	1.05 (0.93–1.17)	1.11 (0.99–1.24)	1.09 (0.97–1.22)	1.28 (1.14–1.43)	< 0.001
**Outcome of more than 20 hospital days**						
Model 1	1.00	1.09 (1.00–1.19)	1.10 (1.00–1.20)	1.14 (1.04–1.24)	1.27 (1.17–1.39)	< 0.001
Model 1†	1.00	1.08 (0.96–1.22)	1.15 (1.02–1.30)	1.23 (1.09–1.40)	1.45 (1.28–1.64)	< 0.001
Model 2	1.00	1.08 (0.99–1.18)	1.05 (0.96–1.15)	1.09 (1.00–1.19)	1.18 (1.07–1.29)	0.001
Model 2†	1.00	1.06 (0.94–1.21)	1.09 (0.96–1.23)	1.16 (1.03–1.32)	1.34 (1.18–1.51)	< 0.001

Model 1: Adjusted for age and sex. Model 2: Adjusted for age, sex, manual social class, low education, current cigarette smoker, body mass index > 30kg/m2 † Excluding participants who died prior to April 2018

For peer review only

Figure 1 shows graphs of length of hospital stay by quintiles of Townsend index and demonstrates the disparity between individual socioeconomic factors and hospital stay when area deprivation index is also considered. In the first plot, results are stratified by higher and lower educational attainment. The difference in days between the least deprived (quintile 1) and the most deprived (quintile 5) is 6 days for those with lower educational attainment and 3 days for those with higher educational attainment. The second plot shows results stratified by manual and non-manual social class. The difference in days between the least deprived and the most deprived is 8 days for those with a manual social class and 3 days for those with a non-manual social class. Significant interactions were observed between social class, level of education and Townsend index ($p_{\text{Interaction}}$=0.025 and $p_{\text{Interaction}}$=0.020 respectively)

INSERT FIGURE 1 HERE

Supplementary table S1 shows the multivariable logistic regression for risk factors for outcomes of any hospital admissions, ≥7 hospital admissions and >20 days of hospital stay between 1999 to 2018. Models are presented for all participants, men and women and each risk factor is adjusted for all others for the nine models. Male sex is only included in the models for all participants. As well as age, social class, education and body mass index, the four individual components of Townsend index are modelled. As previously reported, age, male sex, lower education level, manual social class, current cigarette smoking and a body mass index >30 kg/m^2 were all associated with increased hospitalisation. No single component of the Townsend index was more strongly associated for all outcomes for both men and women. However, the unemployment component was associated with seven or more hospital admissions. Areas with low car ownership appeared to have a greater association in women than men.

Supplementary table S2 displays logistic regression models for the outcome of more than 20 hospital days for Townsend index in various subgroups. Models are stratified by a dichotomised subgroup: men and women, age above or below 65 years, manual and non-manual social class, lower or higher education level, smoking status, BMI above and below 30 kg/m^2, urban or rural home postcode, and moved house between the year 2000 and 2014. Odds ratios (OR) within all strata were in consistent directions with no interaction by age, smoking status or BMI.

The numbers of individuals with missing values for covariables were: 53 BMI, 218 smoking status, 545 social class, 18 education level.

Discussion

Residential area deprivation was associated with future hospital usage independently of individual sociodemographic factors in particular, age, sex, social class and education, as well as lifestyle factors smoking and body mass index in this cohort of middle-aged and older men and women. Study participants in the highest fifth of the Townsend Index - those living in the most deprived areas, at or below the national average, were more likely to spend more than 20 days in hospital or be admitted to hospital on more than 7 occasions. There were also significant interactions between residential area deprivation and individual social class and education level. Participants with a manual social class living in an area with higher deprivation index spent longer in hospital than those with manual occupations living in less deprived areas. Similarly, those with lower education level living in more deprived areas had the greatest risk of hospitalisation. This suggests that hospitalisation is greatest when those with poorer individual socioeconomic factors are combined with residential deprivation. We considered a number of possible explanations for these findings.

Strengths and limitations of this study

The EPIC-Norfolk cohort is very well characterised. This enabled us to take into account many potentially confounding variables understood to be related to hospital usage and disease. The UK National Health Service is free at the point of use and consequently income is not a major determinant of hospital admissions. Despite this,

For peer review only - http://bmjopen.bmj.com/site/about/guidelines.xhtml

social class, education and residential deprivation were all independently related to hospital use. Our study examines hospital activity using a prospective cohort design in a population of community-dwelling participants with clearly defined population denominators. It uses a large cohort of middle aged and older men and women with 19 years of follow-up time having both area based census measures and individual social class and education level from questionnaires available.

Townsend Area Deprivation Index is associated with individual sociodemographic factors such as occupational social class and education and other factors including age, sex and BMI. Since all these factors are also related to hospital use, some level of confounding will be present. However, multivariable regression models adjusting for all these variables only modestly attenuated the area deprivation associations. In table S2, we stratified by the main confounders and the results remained consistent in the subgroups. The accuracy of the measurement might not be sufficient to ensure adequate adjustment so we cannot exclude the possibility of residual confounding with known or other unknown factors associated with both Townsend Index and hospital usage. These unknown factors may either attenuate or strengthen the associations. Interactions between area deprivation and individual sociodemographic factors highlighted stronger associations among more deprived groups.

The use of area-based measurements has some limitations. The factors used in the Townsend score may vary in their ability to assess deprivation according to setting. In urban areas, lower car ownership rates may reflect the availability of other transport options and closer proximity of work places and facilities such as shops. In rural areas, overcrowding may be less common while car ownership may be more of a necessity while simultaneously a drain on resources. The deprivation index is based on data from the UK census that only takes place every ten years and over the period under examination, areas may change becoming more or less deprived.

Area deprivation was determined by postcode of residence in the year 2000. Study participants who moved house may have been misclassified for some of the period over which hospitalisation was assessed. However, while 22% of the cohort moved house between the years 2000 and 2014, the large majority of participants relocated locally in Norfolk, with others moving elsewhere in England and Wales. Since the Townsend Index was not measured at enumeration district level in the UK census beyond 1991, no directly comparable measure was available at later time points to examine change. However, a sensitivity analysis of non-movers found very similar results to the main analyses and any misclassification due to moves or changes over time in residential area deprivation scoring and resultant measurement error would only be likely to attenuate associations with the residential area score. HES record were available for participants who relocated within England and Wales and hence there was virtually no loss to follow-up.

Differential misclassification in hospital use may be explained by early death rates. Study participants living in more deprived areas may have died earlier and not used hospital services for the full period. However, while the death rate was higher among those living in the most deprived areas, 65% of the cohort survived beyond 2018 and models restricted to survivors were more strongly associated with outcome measures than those in the main analysis. Sociodemographic factors may be less relevant for the very seriously ill who require hospital treatment at the end of life.

It may also be possible that individuals did not use NHS facilities but private hospitals differently by socioeconomic status which might explain lower use in the higher sociodemographic groups. However, the use of private hospitals in the Norfolk area over this time period was minimal [27] and hence record linkage of routinely collected hospital episode data gave virtually complete ascertainment. Reverse causation is also possible whereby those in poor health at recruitment may have lower occupational social class increasing the chance of them living in a more deprived area. However, hospitalisation rates were low in the period directly after recruitment.

Comparison with other studies

Inequality in healthcare favouring the better off has been observed in many countries [10–12] and healthcare insurance and eligibility for government healthcare based on income thresholds may influence the associations

For peer review only - http://bmjopen.bmj.com/site/about/guidelines.xhtml

observed. NHS healthcare is not constrained by ability to pay and hence we were able to examine the independent association of residential area deprivation - material living conditions, poor quality housing and poor infrastructure - and its association with subsequent hospitalisation.

There is some evidence to suggest that travel time is associated with hospital use [31,32] but there was no strong association in this study. Study participants were approximately evenly divided into those living in urban and those in rural areas. The moderately deprived (those with Townsend quintile 2-4) were more likely to live in rural areas while the most deprived (Townsend quintile 5) were predominantly urban dwellers. Study participants in Townsend quintiles 1 and 5 were closer by road from their home to the Norfolk and Norwich hospital but the time taken for the journey did not vary greatly. Neither distance from hospital nor urban or rural location explained our findings, since those in the lowest deprivation areas are mainly urban with the shortest travel time to hospital. Studies examining urban/rural populations and car ownership have noted differences in deprivation characteristics. [33,34] However, irrespective of travel distance or time, owning or having access to a car would make a considerable difference in being able to access local facilities. Although there may be more regular public transport services in cities, this will vary and cost and limited travel options may restrict access not only to hospital but also to friends and relatives, to better quality supermarkets and to parks and recreational facilities.

Most studies examining deprivation in the context of health, disease and mortality either rely on area based measures collected for example from census data [16–20] or from individual level data from questionnaires. [13,14] We had access to both forms of information, having derived individual social class and education level from self reported questionnaires and area level measures from residential postcode linkage. Hospital based studies using patients as study participants do not have a reliable population based denominator and cannot estimate overall risk in the population. Studies often attempt to define a denominator using separate population estimates while not individually linking. [16,35,36] We were able to examine hospital usage over 19 years in a clearly defined community based population using a prospective cohort design.

Norfolk is an area of generally low deprivation with >80% of the study population living in areas with deprivation levels below the national average. Few participants live in areas of high deprivation such as those found in some larger cities in other parts of the country. Those living in more deprived cities or regions have a socioeconomic gradient in hospital usage more extreme than we were able to observe [37] but while our study does not provide any information on the most extreme forms of deprivation, there was sufficient heterogeneity to observe large differences in hospital use.

Our results provide further evidence adding to the substantial literature linking deprivation to health. Unlike many studies, we used overall measures of hospital activity, including both elective and emergency admissions and found evidence of an independent association of residential area deprivation not accounted for by known individual factors such as social class and education. Our results also demonstrate that the combination of residential area deprivation with lower levels of education or manual social class result in the highest levels of hospitalisation.

The Black report [2,3] concluded that health inequalities were not mainly attributable to failings in the NHS, but rather to many other social inequalities influencing health: income, education, housing, diet, employment, and conditions of work. It suggested two mechanisms for how social determinants influence health: cultural/behavioural and materialist/structuralist. Some authors have pointed out that research on the determinants of health are generally focused on the individual but patterns of population health are unclear without examining structural determinants at the societal level. [38] Townsend's residential deprivation index uses aggregate measures of particular characteristics for people living in an area. It has been used mainly as a surrogate for individual measures of deprivation in many studies. [39] We were not able to examine physical features of the environment in this study. Ecological measurements such as the quality of housing, access to recreational facilities, local services provided, community support and levels of crime may affect health and hospital usage. However, we were able to examine both individual and area level deprivation in the same study participants, and the interaction we observed suggests that there is a higher risk hospitalisation in more deprived

For peer review only

areas of residence disproportionately for those with lower individual social class and education. Conversely, individuals with non-manual social class and higher levels of education appear more resilient to hospitalisation irrespective of the level of deprivation of their residence.

Conclusions and policy implications

There is a socioeconomic gradient in hospital usage for factors measured both individually and at area level. Residential area deprivation predicts future hospitalisations, time spent in hospital and number of admissions, independently of individual social class and education level and other behavioural factors. There are significant interactions such that residential area deprivation has greater impact in those with low education level or manual social class. Conversely, higher education level and social class mitigated the association of area deprivation with hospital usage. Effective NHS and government policy should therefore involve addressing deprivation both at the individual and infrastructural levels to identify and target those most at risk within the community. NHS policies focused on reducing health inequalities in the elderly need to work alongside wider government initiatives to improve the quality of housing, transport and infrastructure and access to recreation and green space.

References

1 Antonovsky A. Social Class and Illness: A Reconsideration. *Sociological Inquiry* 1967;**37**:311–22. doi:10.1111/j.1475-682X.1967.tb00661.x

2 Black D. Inequalities in Health: Report of a Research Working Group. 1980.

3 Acheson D. *Independent inquiry into inequalities in health: report*. HM Stationery Office 1998.

4 Smith GD, Bartley M, Blane D. The Black report on socioeconomic inequalities in health 10 years on. *BMJ* 1990;**301**:373–7.

5 Health expectancies at birth and at age 65 in the UK, based on 2011 Census health and disability prevalence data - Office for National Statistics. https://www.ons.gov.uk/peoplepopulationandcommunity/healthandsocialcare/healthandlifeexpectancies/bulletins/healthexpectanciesatbirthandatage65intheukbasedon2011censushealthanddisabilityprevalencedata/2010to2012 (accessed 23 May 2018).

6 Life Expectancy at Birth and at Age 65 by Local Areas in England and Wales - Office for National Statistics. https://www.ons.gov.uk/peoplepopulationandcommunity/birthsdeathsandmarriages/lifeexpectancies/bulletins/lifeexpectancyatbirthandatage65bylocalareasinenglandandwales/2015-11-04 (accessed 23 May 2018).

7 Marmot M, Friel S, Bell R, *et al.* Closing the gap in a generation: health equity through action on the social determinants of health. *The Lancet* 2008;**372**:1661–9. doi:10.1016/S0140-6736(08)61690-6

8 Stafford M, Marmot M. Neighbourhood deprivation and health: does it affect us all equally? *Int J Epidemiol* 2003;**32**:357–66. doi:10.1093/ije/dyg084

9 Townsend P, Phillimore P, Beattie A. Health and deprivation. Inequality and the North. *Croom Helm Ltd, London* Published Online First: 1987. doi:10.1016/0168-8510(88)90006-1

10 Doorslaer E van, Masseria C, Koolman X. Inequalities in access to medical care by income in developed countries. *CMAJ* 2006;**174**:177–83. doi:10.1503/cmaj.050584

11 Pickett KE, Wilkinson RG. Income inequality and health: A causal review. *Social Science & Medicine* 2015;**128**:316–26. doi:10.1016/j.socscimed.2014.12.031

12 Fjær EL, Balaj M, Stornes P, *et al.* Exploring the differences in general practitioner and health care specialist utilization according to education, occupation, income and social networks across Europe: findings from the

European social survey (2014) special module on the social determinants of health. *Eur J Public Health* 2017;**27**:73–81. doi:10.1093/eurpub/ckw255

13 Head J, Chungkham HS, Hyde M, *et al.* Socioeconomic differences in healthy and disease-free life expectancy between ages 50 and 75: a multi-cohort study. *Eur J Public Health* 2019;**29**:267–72. doi:10.1093/eurpub/cky215

14 McFadden E, Luben R, Wareham N, *et al.* Occupational social class, risk factors and cardiovascular disease incidence in men and women: a prospective study in the European Prospective Investigation of Cancer and Nutrition in Norfolk (EPIC-Norfolk) cohort. *Eur J Epidemiol* 2008;**23**:449. doi:10.1007/s10654-008-9262-2

15 Luben R, Hayat S, Wareham N, *et al.* Predicting admissions and time spent in hospital over a decade in a population-based record linkage study: the EPIC-Norfolk cohort. *BMJ Open* 2016;**6**:e009461. doi:10.1136/bmjopen-2015-009461

16 Asaria M, Doran T, Cookson R. The costs of inequality: whole-population modelling study of lifetime inpatient hospital costs in the English National Health Service by level of neighbourhood deprivation. *J Epidemiol Community Health* 2016;**70**:990–6. doi:10.1136/jech-2016-207447

17 Bernard S, Smith LK. Emergency admissions of older people to hospital: a link with material deprivation. *J Public Health (Oxf)* 1998;**20**:97–101. doi:10.1093/oxfordjournals.pubmed.a024727

18 Conway R, Galvin S, Coveney S, *et al.* Deprivation as an outcome determinant in emergency medical admissions. *QJM* 2013;**106**:245–51. doi:10.1093/qjmed/hcs233

19 Conway R, Byrne D, O'Riordan D, *et al.* Deprivation index and dependency ratio are key determinants of emergency medical admission rates. *European Journal of Internal Medicine* 2015;**26**:709–13. doi:10.1016/j.ejim.2015.09.010

20 Moore L, Cisse B, Batomen Kuimi BL, *et al.* Impact of socio-economic status on hospital length of stay following injury: a multicenter cohort study. *BMC Health Serv Res* 2015;**15**. doi:10.1186/s12913-015-0949-2

21 Macintyre S, Maciver S, Sooman A. Area, Class and Health: Should we be Focusing on Places or People? *Journal of Social Policy* 1993;**22**:213. doi:10.1017/S0047279400019310

22 Smith GD, Hart C, Watt G, *et al.* Individual social class, area-based deprivation, cardiovascular disease risk factors, and mortality: the Renfrew and Paisley Study. *J Epidemiol Community Health* 1998;**52**:399–405.

23 Shohaimi S, Luben R, Wareham N, *et al.* Residential area deprivation predicts smoking habit independently of individual educational level and occupational social class. A cross sectional study in the Norfolk cohort of the European Investigation into Cancer (EPIC-Norfolk). *Journal of Epidemiology & Community Health* 2003;**57**:270–6. doi:10.1136/jech.57.4.270

24 Hayat SA, Luben R, Keevil VL, *et al.* Cohort Profile: A prospective cohort study of objective physical and cognitive capability and visual health in an ageing population of men and women in Norfolk (EPIC-Norfolk 3). *Int J Epidemiol* 2014;**43**:1063–72. doi:10.1093/ije/dyt086

25 Day N, Oakes S, Luben R, *et al.* EPIC-Norfolk: study design and characteristics of the cohort. European Prospective Investigation of Cancer. *Br J Cancer* 1999;**80 Suppl 1**:95–103.

26 Phillimore P, Beattie A, Townsend P. Widening inequality of health in northern England, 1981-91. *BMJ* 1994;**308**:1125–8.

27 Boyle S. United Kingdom (England): Health system review. *Health Syst Transit* 2011;**13**:1–483, xix–xx.

28 Bland R. Measuring "Social Class": A Discussion of the Registrar-General's Classification. *Sociology* 1979;**13**:283–91. doi:10.1177/003803857901300209

29 McFadden E, Luben R, Khaw K-T. Different measures of social class in women and mortality. *Eur J Epidemiol* 2009;**24**:231–6. doi:10.1007/s10654-009-9332-0

For peer review only

30 Luxen D, Vetter C. Real-time routing with OpenStreetMap data. In: *Proceedings of the 19th ACM SIGSPATIAL International Conference on Advances in Geographic Information Systems*. Chicago, Illinois: : ACM 2011. 513–6. doi:10.1145/2093973.2094062

31 Kelly C, Hulme C, Farragher T, *et al.* Are differences in travel time or distance to healthcare for adults in global north countries associated with an impact on health outcomes? A systematic review. *BMJ Open* 2016;**6**:e013059. doi:10.1136/bmjopen-2016-013059

32 Nicholl J, West J, Goodacre S, *et al.* The relationship between distance to hospital and patient mortality in emergencies: an observational study. *Emerg Med J* 2007;**24**:665–8. doi:10.1136/emj.2007.047654

33 Gilthorpe MS, Wilson RC. Rural/urban differences in the association between deprivation and healthcare utilisation. *Social Science & Medicine* 2003;**57**:2055–63. doi:10.1016/S0277-9536(03)00071-6

34 Christie SML, Fone DL. Does car ownership reflect socio-economic disadvantage in rural areas? A cross-sectional geographical study in Wales, UK. *Public Health* 2003;**117**:112–6. doi:10.1016/S0033-3506(02)00027-6

35 Billings J, Georghiou T, Blunt I, *et al.* Choosing a model to predict hospital admission: an observational study of new variants of predictive models for case finding. *BMJ Open* 2013;**3**:e003352. doi:10.1136/bmjopen-2013-003352

36 Gray DP, Henley W, Chenore T, *et al.* What is the relationship between age and deprivation in influencing emergency hospital admissions? A model using data from a defined, comprehensive, all-age cohort in East Devon, UK. *BMJ Open* 2017;**7**:e014045. doi:10.1136/bmjopen-2016-014045

37 Macintyre S, Macdonald L, Ellaway A. Do poorer people have poorer access to local resources and facilities? The distribution of local resources by area deprivation in Glasgow, Scotland. *Social Science & Medicine* 2008;**67**:900–14. doi:10.1016/j.socscimed.2008.05.029

38 Lynch JW, Kaplan GA. Understanding How Inequality in the Distribution of Income Affects Health. *J Health Psychol* 1997;**2**:297–314. doi:10.1177/135910539700200303

39 Macintyre S, Maciver S, Sooman A. Area, Class and Health: Should we be Focusing on Places or People? *Journal of Social Policy* 1993;**22**:213–34. doi:10.1017/S0047279400019310

For peer review only

Supporting information

Supplementary Table S1 | Multivariable logistic regression of risk factors for any hospital admissions, ≥7 hospital admissions and >20 days of hospital stay from 1999 to 2018 in 24 977 men and women. Townsend index components using the UK 1991 census

Supplementary Table S2 | Multivariable logistic regression of Townsend Index and more than twenty hospital days in subgroups

Figure 1: Hospitalisation by deprivation index. Length of hospital stay over 19 years of follow-up by quintiles of Townsend Index grouped by categories of education level and categories of social class. Low education level is defined as those having no qualifications and high education as those with at least some qualifications at secondary level or above. Length of stay is truncated to 365 days for those staying longer than 365 days. Interaction tested using multivariable adjusted linear regression with covariables age, sex, education level (higher/lower), body mass index (≤30/>30) smoking status (current/non-current)

For peer review only

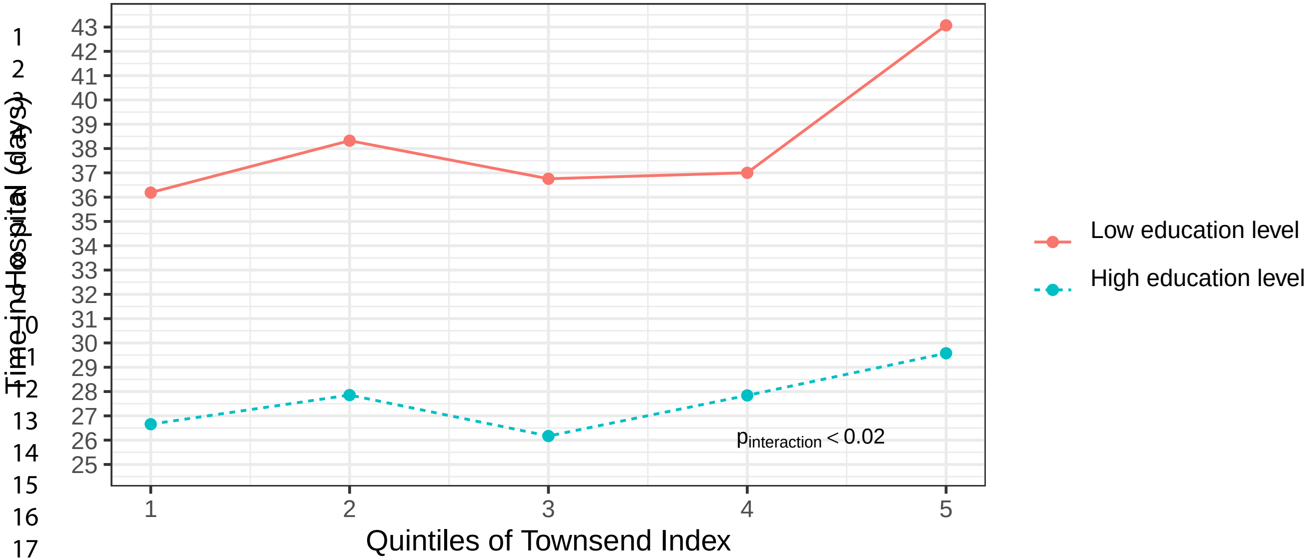

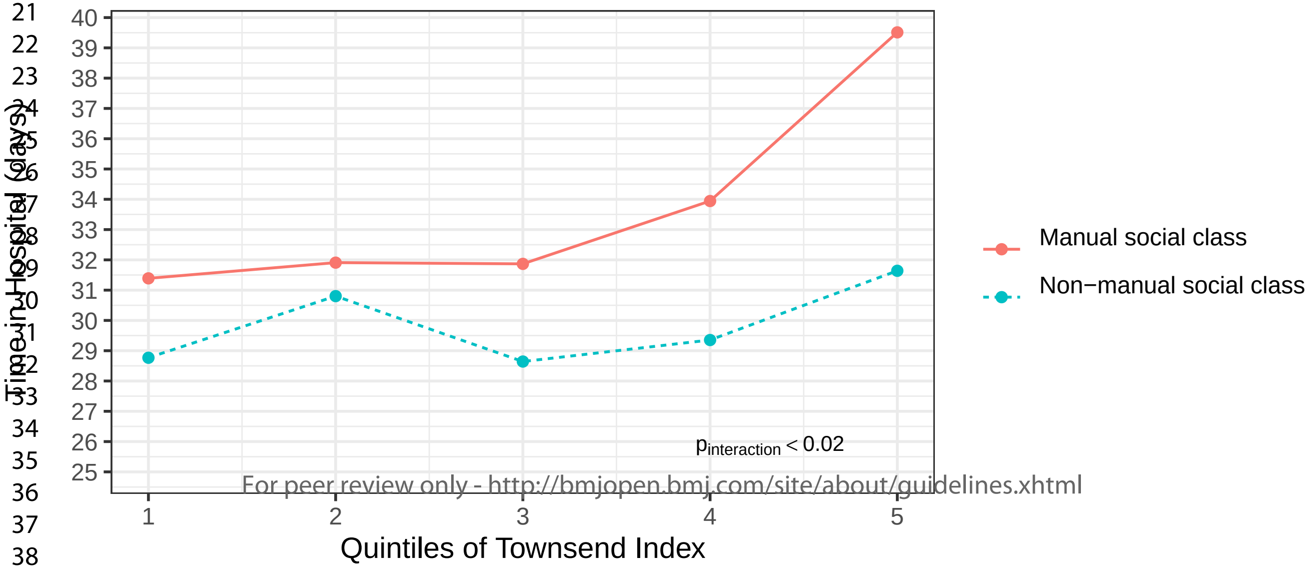

For peer review only - http://bmjopen.bmj.com/site/about/guidelines.xhtml

Supplementary Table S1 | Multivariable logistic regression of risk factors for any hospital admissions, ≥7 hospital admissions and >20 days of hospital stay from 1999 to 2018 in 24 977 men and women. Townsend index components using the UK 1991 census

	All subjects OR (95% CI)	p value	Men OR (95% CI)	p value	Women OR (95% CI)	p value
**Outcome of any hospital admissions**						
Male sex	1.13 (1.04–1.23)	0.006				
Age per 10 years	2.08 (1.98–2.20)	< 0.001	2.15 (1.99–2.34)	< 0.001	2.03 (1.89–2.17)	< 0.001
Unemployment Z-Score >0	1.06 (0.92–1.21)	0.426	1.06 (0.87–1.31)	0.560	1.05 (0.88–1.26)	0.604
Households with no car Z-Score >0	1.05 (0.89–1.23)	0.568	0.91 (0.72–1.16)	0.459	1.16 (0.94–1.44)	0.161
Households not owner-occupied Z-Score >0	1.03 (0.90–1.19)	0.625	0.99 (0.81–1.22)	0.948	1.07 (0.89–1.28)	0.479
Household overcrowding Z-Score >0	0.98 (0.88–1.10)	0.760	1.00 (0.84–1.19)	0.983	0.97 (0.84–1.13)	0.717
Manual social class	1.16 (1.06–1.27)	0.001	1.25 (1.08–1.43)	0.002	1.11 (0.98–1.25)	0.106
Low education level	1.35 (1.22–1.50)	< 0.001	1.21 (1.03–1.43)	0.026	1.45 (1.27–1.66)	< 0.001
Current smoker	1.21 (1.06–1.38)	0.006	1.15 (0.94–1.41)	0.174	1.26 (1.05–1.51)	0.013
BMI>30 kg/m^2	1.28 (1.12–1.46)	< 0.001	1.19 (0.97–1.47)	0.095	1.34 (1.13–1.59)	< 0.001
**Outcome of 7 or more hospital admissions**						
Male sex	1.23 (1.16–1.30)	< 0.001				
Age per 10 years	1.49 (1.44–1.54)	< 0.001	1.50 (1.43–1.57)	< 0.001	1.48 (1.42–1.55)	< 0.001
Unemployment Z-Score >0	1.18 (1.08–1.28)	< 0.001	1.17 (1.04–1.33)	0.012	1.18 (1.06–1.33)	0.004
Households with no car Z-Score >0	0.98 (0.88–1.08)	0.641	0.92 (0.79–1.06)	0.253	1.03 (0.90–1.17)	0.692
Households not owner-occupied Z-Score >0	0.93 (0.86–1.02)	0.134	0.91 (0.80–1.04)	0.163	0.95 (0.85–1.08)	0.447
Household overcrowding Z-Score >0	1.08 (1.00–1.16)	0.044	1.07 (0.96–1.19)	0.207	1.08 (0.98–1.20)	0.112
Manual social class	1.15 (1.08–1.22)	< 0.001	1.16 (1.06–1.26)	< 0.001	1.14 (1.06–1.24)	0.001
Low education level	1.14 (1.07–1.21)	< 0.001	1.12 (1.02–1.23)	0.014	1.15 (1.06–1.25)	< 0.001
Current smoker	1.34 (1.23–1.46)	< 0.001	1.25 (1.10–1.41)	< 0.001	1.44 (1.28–1.61)	< 0.001
BMI>30 kg/m^2	1.38 (1.28–1.49)	< 0.001	1.39 (1.24–1.56)	< 0.001	1.38 (1.25–1.52)	< 0.001
**Outcome of more than 20 hospital days**						
Male sex	1.16 (1.10–1.23)	< 0.001				
Age per 10 years	2.68 (2.59–2.78)	< 0.001	2.62 (2.49–2.76)	< 0.001	2.73 (2.61–2.87)	< 0.001
Unemployment Z-Score >0	1.10 (1.01–1.20)	0.034	1.10 (0.96–1.25)	0.161	1.10 (0.98–1.24)	0.111
Households with no car Z-Score >0	1.00 (0.90–1.11)	0.979	0.95 (0.81–1.11)	0.501	1.04 (0.90–1.19)	0.599
Households not owner-occupied Z-Score >0	1.00 (0.92–1.10)	0.916	1.01 (0.88–1.16)	0.849	1.00 (0.88–1.13)	0.958
Household overcrowding Z-Score >0	1.07 (0.99–1.15)	0.100	1.03 (0.92–1.15)	0.622	1.10 (0.99–1.22)	0.071
Manual social class	1.16 (1.09–1.24)	< 0.001	1.19 (1.09–1.31)	< 0.001	1.14 (1.05–1.24)	0.003
Low education level	1.16 (1.09–1.24)	< 0.001	1.18 (1.07–1.30)	< 0.001	1.15 (1.05–1.25)	0.001
Current smoker	1.57 (1.44–1.72)	< 0.001	1.61 (1.41–1.83)	< 0.001	1.54 (1.37–1.74)	< 0.001
BMI>30 kg/m^2	1.61 (1.49–1.74)	< 0.001	1.58 (1.40–1.79)	< 0.001	1.63 (1.48–1.81)	< 0.001

Supplementary Table S2 | Multivariable logistic regression of Townsend Index and more than twenty hospital days in subgroups

	Townsend Index OR (95% CI)†
**Men and women**	
Men (n=11214)	1.01 (0.99–1.03)
Women (n=13763)	1.03 (1.01–1.05)
**By age above and below 65 years**	
Less than 65 years (n=17343)	1.03 (1.01–1.05)
65 years and above (n=7634)	1.01 (0.98–1.03)
**Manual and non-manual social class**	
Non-manual (n=14691)	1.02 (1.00–1.04)
Manual (n=9741)	1.03 (1.00–1.05)
**By level of education**	
Higher level (n=15841)	1.01 (0.99–1.03)
Lower level (n=9118)	1.03 (1.01–1.06)
**By smoking status**	
Former or never smoker (n=21864)	1.02 (1.01–1.04)
Current smoker (n=2895)	1.00 (0.97–1.04)
**By level of body mass index**	
BMI ≤ 30 kg/m² (n=21124)	1.02 (1.01–1.04)
BMI > 30 kg/m² (n=3800)	1.01 (0.98–1.04)
**Urban or rural home postcode**	
Urban (n=11214)	1.01 (1.00–1.03)
Rural (n=13763)	1.03 (1.01–1.06)
**Moved house between 2000 and 2014**	
Moved house (n=5355)	1.04 (1.01–1.07)
Did not move house (n=18728)	1.01 (1.00–1.03)

Logistic regression per unit Townsend Index. † Adjusted for age, current smoking, BMI (categories)

STROBE Statement—Checklist of items that should be included in reports of ***cohort studies***

	Item No	Recommendation	Page No
**Title and abstract**	1	(*a*) Indicate the study's design with a commonly used term in the title or the abstract	1
		(*b*) Provide in the abstract an informative and balanced summary of what was done and what was found	2
**Introduction**			
Background/rationale	2	Explain the scientific background and rationale for the investigation being reported	4
Objectives	3	State specific objectives, including any prespecified hypotheses	4
**Methods**			
Study design	4	Present key elements of study design early in the paper	5 and title
Setting	5	Describe the setting, locations, and relevant dates, including periods of recruitment, exposure, follow-up, and data collection	5
Participants	6	(*a*) Give the eligibility criteria, and the sources and methods of selection of participants. Describe methods of follow-up	6
		(*b*) For matched studies, give matching criteria and number of exposed and unexposed	
Variables	7	Clearly define all outcomes, exposures, predictors, potential confounders, and effect modifiers. Give diagnostic criteria, if applicable	6
Data sources/ measurement	8*	For each variable of interest, give sources of data and details of methods of assessment (measurement). Describe comparability of assessment methods if there is more than one group	5-6
Bias	9	Describe any efforts to address potential sources of bias	10-11
Study size	10	Explain how the study size was arrived at	6
Quantitative variables	11	Explain how quantitative variables were handled in the analyses. If applicable, describe which groupings were chosen and why	6
Statistical methods	12	(*a*) Describe all statistical methods, including those used to control for confounding	6
		(*b*) Describe any methods used to examine subgroups and interactions	Table S2

For peer review only - http://bmjopen.bmj.com/site/about/guidelines.xhtml

		(*c*) Explain how missing data were addressed	6
		(*d*) If applicable, explain how loss to follow-up was addressed	11
		(*e*) Describe any sensitivity analyses	10
**Results**			
Participants	13*	(a) Report numbers of individuals at each stage of study—eg numbers potentially eligible, examined for eligibility, confirmed eligible, included in the study, completing follow-up, and analysed	5-6
		(b) Give reasons for non-participation at each stage	6
		(c) Consider use of a flow diagram	
Descriptive data	14*	(a) Give characteristics of study participants (eg demographic, clinical, social) and information on exposures and potential confounders	Table 1
		(b) Indicate number of participants with missing data for each variable of interest	9
		(c) Summarise follow-up time (eg, average and total amount)	Stated in title
Outcome data	15*	Report numbers of outcome events or summary measures over time	Table 1

Main results	16	(*a*) Give unadjusted estimates and, if applicable, confounder-adjusted estimates and their precision (eg, 95% confidence interval). Make clear which confounders were adjusted for and why they were included	8, table 2
		(*b*) Report category boundaries when continuous variables were categorized	6 (Statistical analysis)
		(*c*) If relevant, consider translating estimates of relative risk into absolute risk for a meaningful time period	
Other analyses	17	Report other analyses done—eg analyses of subgroups and interactions, and sensitivity analyses	9, Table S2, Covariables in subgroups
**Discussion**			
Key results	18	Summarise key results with reference to study objectives	10
Limitations	19	Discuss limitations of the study, taking into account sources of potential bias or imprecision. Discuss both direction and magnitude of any potential bias	10-11
Interpretation	20	Give a cautious overall interpretation of results considering objectives, limitations, multiplicity of analyses, results from similar studies, and other relevant evidence	12
Generalisability	21	Discuss the generalisability (external validity) of the study results	12
**Other information**			
Funding	22	Give the source of funding and the role of the funders for the present study and, if applicable, for the original study on which the present article is based	

*Give information separately for exposed and unexposed groups.

Note: An Explanation and Elaboration article discusses each checklist item and gives methodological background and published examples of transparent reporting. The STROBE checklist is best used in conjunction with this article (freely available on the Web sites of PLoS Medicine at http://www.plosmedicine.org/, Annals of Internal Medicine at http://www.annals.org/, and Epidemiology at http://www.epidem.com/). Information on the STROBE Initiative is available at http://www.strobe-statement.org.

BMJ Open

Residential area deprivation and risk of subsequent hospital admission in a British population: the EPIC-Norfolk cohort

Journal:	*BMJ Open*
Manuscript ID	bmjopen-2019-031251.R2
Article Type:	Research
Date Submitted by the Author:	12-Nov-2019
Complete List of Authors:	Luben, Robert; University of Cambridge School of Clinical Medicine, Department of Public Health and Primary Care<br>Hayat, Shabina; University of Cambridge School of Clinical Medicine, Department of Public Health and Primary Care<br>Khawaja, Anthony; Moorfields Eye Hospital, NIHR Biomedical Research Centre<br>Wareham, Nicholas; University of Cambridge School of Clinical Medicine, MRC Epidemiology Unit<br>Pharoah, Paul; University of Cambridge School of Clinical Medicine, Department of Public Health and Primary Care<br>Khaw, Kay-Tee; University of Cambridge School of Clinical Medicine, Department of Public Health and Primary Care
**Primary Subject Heading**:	Epidemiology
Secondary Subject Heading:	Health services research, Health policy
Keywords:	EPIDEMIOLOGY, Health policy < HEALTH SERVICES ADMINISTRATION & MANAGEMENT, PUBLIC HEALTH, SOCIAL MEDICINE

SCHOLARONE™
Manuscripts

Residential area deprivation and risk of subsequent hospital admission in a British population: the EPIC-Norfolk cohort

Robert Luben[1], Shabina Hayat[1], Anthony Khawaja[2], Nicholas Wareham[3], Paul Pharoah[1] , Kay-Tee Khaw[1]

1 Department of Public Health and Primary Care, Institute of Public Health, University of Cambridge, Cambridge, United Kingdom
2 NIHR Biomedical Research Centre, Moorfields Eye Hospital NHS Foundation Trust and UCL Institute of Ophthalmology, London, UK
3 MRC Epidemiology Unit, University of Cambridge School of Clinical Medicine, Cambridge United Kingdom
Correspondence to: Robert Luben, Department of Public Health and Primary Care, Strangeways Research Laboratory, Worts Causeway, Cambridge, UK. Email: robert.luben@phpc.cam.ac.uk Telephone: +44 (0)1223 748691

For peer review only

Word count: 4432

October 17, 2019

ABSTRACT

OBJECTIVES

To investigate whether residential area deprivation index predicts subsequent admissions to hospital and time spent in hospital independently of individual social class and lifestyle factors.

DESIGN

Prospective population based study.

SETTING

The EPIC-Norfolk Study.

PARTICIPANTS

11 214 men and 13 763 women in the general population, aged 40-79 years at recruitment (1993 to 1997), alive in 1999.

MAIN OUTCOME MEASURE

Total admissions to hospital and time spent in hospital during a 19-year time period (1999 to 2018).

RESULTS

Compared to those with residential Townsend Area Deprivation Index lower than the average for England and Wales, those with a higher than average deprivation index had a higher likelihood of spending more than twenty days in hospital multivariable adjusted odds ratio (OR) 1.18 (95% confidence interval (CI) 1.07 to 1.29) and having 7 or more admissions OR 1.11 (95% CI 1.02 to 1.22) after adjustment for age, sex, smoking status, education, social class, and body mass index. Occupational social class and educational attainment modified the association between area deprivation and hospitalisation; those with manual social class and lower education level were at greater risk of hospitalisation when living in an area with higher deprivation index ($p_{Interaction}$=0.025 and $p_{Interaction}$=0.020 respectively), while the risk for non-manual and more highly educated participants did not vary greatly by area of residence.

CONCLUSION

Residential area deprivation predicts future hospitalisations, time spent in hospital and number of admissions, independently of individual social class and education level and other behavioural factors. There are significant interactions such that residential area deprivation has greater impact in those with low education level or manual social class. Conversely, higher education level and social class mitigated the association of area deprivation with hospital usage.

STRENGTHS AND LIMITATIONS OF THIS STUDY

- This study is able to examine hospital activity using a prospective cohort design in a population of community-dwelling participants with clearly defined population denominators.
- It uses a large cohort of middle-aged and older men and women with 19 years of follow-up time and detailed measurements of demographic and behavioural indicators.
- Both area based census measures and individual social class and education level from questionnaires are used.
- Differential misclassification in hospital use may be explained by early death rates.
- Socioeconomic determinants of hospitalisation were examined in the context of UK National Health Service hospitals which are free at the point of use and so not directly influenced by income.

Acknowledgements: The authors would like to thank the participants, general practitioners, and staff of EPIC-Norfolk.

Contributors: KTK,NW SH and RL were involved in the conception and design of the study. RL drafted the manuscript, with support from KTK and PP. AK contributed to data interpretation. RL was responsible for external data linkage. SH and RL contributed to data collection and acquisition. All authors read and critically revised the manuscript and approved the final manuscript. RL is the guarantor. The corresponding author attests that all listed authors meet authorship criteria and that no others meeting the criteria have been omitted.

Funding: EPIC-Norfolk infrastructure and core functions are supported by grants from the Medical Research Council (G0401527) and Cancer Research UK (C864/A8257).

Competing interests: All authors have completed the ICMJE uniform disclosure form at www.icmje.org/coi_disclosure.pdf and report: RL,SH,KTK and NW report grants from MRC and CRUK during the conduct of the study; no support from any organisation for the submitted work; no financial relationships with any organisations that might have an interest in the submitted work in the previous three years; no other relationships or activities that could appear to have influenced the submitted work.

Ethical approval: The work was approval by the East Norfolk and Waveney NHS research governance committee (2005EC07L) and the Norfolk research ethics committee (05/Q0101/191). All participants gave informed signed consent for study participation including access to medical records.

Data sharing: The authors will make the dataset available under a Data Transfer Agreement to any bona fide researcher who wishes to obtain the dataset in order to undertake a replication analysis. Although the dataset is anonymised, the breadth of the data included and the multiplicity of variables that are included in this analysis file as primary variables or confounding factors, means that provision of the dataset to other researchers without a Data Transfer Agreement would constitute a risk.

Transparency: The lead author (the manuscript's guarantor) affirms that the manuscript is an honest, accurate, and transparent account of the study being reported; that no important aspects of the study have been omitted; and that any discrepancies from the study as planned (and, if relevant, registered) have been explained.

Open access: This is an Open Access article distributed in accordance with the terms of the Creative Commons Attribution (CC BY 4.0) license, which permits others to distribute, remix, adapt and build upon this work, for commercial use, provided the original work is properly cited. See: http://creativecommons.org/licenses/by/4.0/

Patient and public involvement: The EPIC-Norfolk Study have an active Participants Advisory Panel which meets quarterly to advise on research protocols, suggest ideas and provide feedback on the research including proposed new studies and collaborations. All participants of the EPIC-Norfolk study are informed about the study through regular newsletters as well as public meetings. Information is also disseminated through local community talks in the Norfolk area and science festivals.

Exclusive licence: The Corresponding Author has the right to grant on behalf of all authors and does grant on behalf of all authors, a worldwide licence to the Publishers and its licensees in perpetuity, in all forms, formats and media (whether known now or created in the future), to i) publish, reproduce, distribute, display and store the Contribution, ii) translate the Contribution into other languages, create adaptations, reprints, include within collections and create summaries, extracts and/or, abstracts of the Contribution, iii) create any other derivative work(s) based on the Contribution, iv) to exploit all subsidiary rights in the Contribution, v) the inclusion of electronic links from the Contribution to third party material where-ever it may be located; and, vi) licence any third party to do any or all of the above.

The Corresponding Author has the right to grant on behalf of all authors and does grant on behalf of all authors, an exclusive licence (or non exclusive for government employees) on a worldwide basis to the BMJ Publishing Group Ltd to permit this article (if accepted) to be published in BMJ editions and any other BMJPGL products and sublicences such use and exploit all subsidiary rights, as set out in our licence.

For peer review only

Introduction

The considerable differences in mortality by social class are well documented [1–4] with those in higher social classes having a typical life expectancy several years longer than those with the lowest. Similarly, life expectancy and health expectancy varies between UK cities and regions with large variations in expected years of life in good health. [5,6] Despite increasing overall life expectancy, inequality remains with lower life and health expectancy observed more often in disadvantaged groups. While lifestyle factors may account for some part of this, the reported differences in death rates cannot be explained by individual behaviour alone. [7,8] Material deprivation was defined by Townsend as 'a state of observable and demonstrable disadvantage relative to the local community or wider society … to which an individual, family or group belongs'. Deprivation indices use factors such as unemployment, the standard of housing, overcrowding and rates of car ownership which together can assess the level of deprivation within a neighbourhood. [9]

Hospitalisation can be measured using the frequency of admission or the length of stay. When measured over a period of time, the outcome represents burden of resources that might be attributable to a population. Inequality in healthcare utilisation favouring patients who are better off is apparent in half of the OECD countries. [10–12] The UK National Health Service is free at the point of use and consequently should provide equitable healthcare not constrained by ability to pay.

Socioeconomic determinants of hospitalisation have been examined using individual level exposures such as occupational social class, income and education and at area level using various deprivation indices but few studies have both individual and area based measures. Individual occupational social class, income and level of education have all been reported to be associated with chronic disease risk. [13,14] We previously reported that a range of simple demographic and behavioural indicators are related to the future probability of cumulative hospital admissions and bed days. [15] Increasing age and male sex and the modifiable factors current cigarette smoking, body mass index (BMI) >30 kg/m^2, manual social class and low education level were all associated with higher future hospital usage over a ten-year period. Area based deprivation measures, available routinely in the UK using postal code linkage, have also reported associations with hospital usage. [16–20] However, the participants in such studies are often limited to those attending hospital and so a suitable population denominator is lacking. Studies reporting health associations for both individual and area measures are less common [21–23] and we are unaware of any studies examining the independent association of residential area deprivation on subsequent hospital usage.

In this paper we examine residential area deprivation using the Townsend deprivation index with subsequent hospital usage over a 19-year period. We explore the independent contribution of residential area deprivation in men and women participants of the EPIC-Norfolk study and its association with future hospitalisation after allowing for the individual level factors previously shown to be associated. We also examine possible interactions between area and individual deprivation measures. Our aim is to determine whether factors such as material living conditions, poor quality housing and poor infrastructure are associated with subsequent hospitalisation in a setting where access to healthcare is unconstrained by ability to pay.

Methods

We used data collected as part of the European Prospective Investigation into Cancer in Norfolk (EPIC-Norfolk), a general population cohort.

For peer review only - http://bmjopen.bmj.com/site/about/guidelines.xhtml

Study design

EPIC-Norfolk is a cohort of men and women living in Norfolk. Recruitment took place between 1993 and 1997 at 35 general practices with invitations sent to all those registered with the practices aged 40–79 years. The design and recruitment of the study has been previously described in detail. [24,25] Briefly, a total of 77 630 invitations were sent to adults registered at participating GP practices; 30 445 (40%) consented to participate in the study of whom 25 639 men and women completed a lifestyle questionnaire and attended a health examination. The study has ethics committee approval from Norfolk Research Ethics Committee (Rec Ref: 98CN01) and all participants gave informed signed consent for study participation including access to medical records. Residential postcode, recorded at the end of recruitment, was used to link to the UK 1991 national census data. [26] Between 1999 and 2018 the cohort was linked to databases maintained by the East Norfolk Primary Health Care Trust and later to national databases held by NHS Digital. [27] Hospital Episode Statistics (HES) records which included admission and discharge dates were used to calculate time in hospital and numbers of admissions. Contiguous admissions were merged and counted as a single admission. Details of linkage of the EPIC-Norfolk cohort participants to hospital records have been previously reported. [15]

Residential area deprivation score for participants

The Townsend Index is an area deprivation measurement calculated using four components: the percentage unemployed of economically active residents over 16 years old, the percentage of households with no car, the percentage of households not owner occupied, and the percentage of households with more than one person per room. These are respectively: a measure of lack of material resources and insecurity, a proxy for current income, a proxy for current wealth, and a measure of material living conditions. The index used in this study was constructed using data collected at the 1991 UK census, which takes place every ten years. Each Townsend component was calculated at Enumeration District (ED), a small area containing an average 175 households used by the census administrators both as output areas and for data collection. Townsend components were then standardised as z-scores at ED level for England and Wales. Study participants were linked to an ED using their home postcode at the end of recruitment in the year 2000. The link was then used to establish a residential Townsend Index for each individual.

Covariables

Participants' height and weight were measured in light clothing without shoes by trained nurses in a clinic setting as part of a health examination between 1993 and 1997. Height was measured to the nearest 0.1 cm using a stadiometer (Chasemores, UK) and weight to the nearest 0.1 kg BMI was calculated using measured weight in kilograms divided by measured height^2 in square metres.

Participants completed a lifestyle questionnaire which included questions about their and their partner's current and past employment. Occupational social class was defined according to the Registrar General's classification [28] and dichotomised into non-manual and manual social classes. Professional, managerial and technical and non-manual skilled occupations (codes I, II and IIIa respectively) were classed as non-manual while manual skilled, partly skilled and unskilled (codes IIIb, IV and V respectively) were classed as manual. Social class for men used (in order of priority) their own current employment, own past employment, partner's current employment or partner's past employment according to whether a social class classification could be defined for a given occupation type. Similarly, social class for women used (in order of priority) their partner's current employment, partner's past employment, own current employment, own past employment. The use of partner's social class for women in the EPIC-Norfolk cohort born between 1918 and 1948 has been previously discussed. [29]

The question "Do you have any of the following qualifications" together with a list of common UK qualifications was used to establish educational attainment. Participants were categorised according to the highest qualification they attained: those with no formal qualifications, those with formal qualifications usually associated with

For peer review only - http://bmjopen.bmj.com/site/about/guidelines.xhtml

completing school aged between 16 and 18 years, and those with degree level qualifications.

Smoking status was derived from two questions: "Have you ever smoked as much as one cigarette a day for as long as a year" and "Do you smoke cigarettes now". The responses to both questions were "yes" or "no" and participants were asked to leave the second question blank if they answered "no" to the first.

Travel time and travel distance between participants home postcode and the Norfolk and Norwich University Hospitals NHS Foundation Trust was calculated using the Open Source Routing Machine (OSRM) [30] which calculates the shortest path between two points over the road network. Postcode of home residence was used to establish if a participant had moved house over the follow-up period. It was available at two points in time: in the year 2000 and the year 2014. Participants whose postcode or house location changed during the period were classified as having moving house but were not excluded from the analyses. Urban and rural categories were established using the 1991 census.

Ascertainment of hospital usage and mortality through record linkage

Details of linkage of the EPIC-Norfolk cohort participants to hospital records have been previously reported. [15] Briefly, linkage using unique NHS numbers was performed between 1999 and 2018 to databases maintained by the East Norfolk Primary Health Care Trust and to national databases held by NHS Digital. [27] All hospital activity for EPIC-Norfolk participants was captured wherever they were treated in England and Wales. Hospital episode statistics (HES) records which included admission and discharge dates were used to calculate time in hospital and numbers of admissions. Contiguous admissions were merged and counted as a single admission.

Statistical analysis

For the current analyses, we excluded the 625 men and women from the baseline cohort who died before 1999. A further 37 who did not have a valid UK postcode were excluded leaving 24 977 participants. Dichotomous variables were created for the three socioeconomic status variables. Occupational social class was categorised into non-manual and manual: social classes I, II, and III non-manual were classified as 'non-manual', while social classes III manual, IV, and V were classified as 'manual'. Educational level was categorised into 'Higher level' (which includes those with qualifications at secondary level or above) and 'Lower level' (those with no qualification). Townsend deprivation index was divided into quintiles. Lower Townsend scores correspond to lower levels of deprivation. Quintiles 1-4 are all below zero and hence below (less deprived than) the national average for England and Wales. Quintile 5 (-0.64, 6.99] corresponds to Townsend scores close to or above the national average (more deprived). Overall Townsend score and components were also dichotomised with scores below zero defined as 'less deprived' and scores above 0 as 'more deprived'. Hospital admissions were categorised into five groups: 0, 1, 2–3, 4–6, and ≥7 while time in hospital was divided into categories: None, Day case, 2–5 days, 6–20 days, and >20 days. The cut-points were chosen to be consistent with earlier work. [15] Since time in hospital was skewed with some people remaining in hospital for extended periods, length of stay longer than 365 days was truncated for graphical presentation. A dichotomous urban/rural variable was defined with "urban" and "urban sparse" as urban and "town", "village" or "hamlet" as rural. Three dichotomous outcome categories were calculated: any hospital admissions (vs no admissions), 7 or more admissions (vs fewer than 7) using total admissions, and greater than 20 hospital days (vs 20 or fewer) using total bed days (overnight stays) and day cases. Multivariable logistic regression was used for all models. All analyses were performed using the R statistical language (R Foundation for Statistical Computing, Vienna, Austria version 3.5.3 with packages knitr, Gmisc, ggplot2, tidyverse, intubate)

Results

Table 1 shows descriptive characteristics by quintiles of residential Townsend Index for 11 214 men and 13 763 women. The majority (n=20 996) of study participants had deprivation index below zero while n=3 981,

For peer review only

approximately corresponding to those in quintile 5, had levels above the national average. Participants in quintile 5 were much more likely to live in an urban setting (70.2%) while those in quintiles 2,3 and 4 were more likely to live in a rural location. Travel distance was lowest for participants in quintile 1 and 5, perhaps due to a higher proportion living in cities and travel times followed a similar pattern. Participants in quintile 5 were the most likely to move house (26.1% between 2000 and 2014). Hospital admissions and time in hospital are shown for both the full cohort and restricted to those who attended hospital. 10.5% of study participants had no admissions over the 19 years from 1999 to 2018.

Table 1 | Descriptive characteristics by quintiles of Townsend Index

	Total	Quintile 1 [-6.74,-3.81]	Quintile 2 (-3.81,-2.94]	Quintile 3 (-2.94,-2.09]	Quintile 4 (-2.09,-0.64]	Quintile 5 (-0.64, 6.99]	P-value
**Sex [n (%)]**							
Men	11214 (44.9)	2271 (45.2)	2262 (45.4)	2280 (45.2)	2226 (45.0)	2175 (43.7)	0.41
Women	13763 (55.1)	2752 (54.8)	2723 (54.6)	2760 (54.8)	2722 (55.0)	2806 (56.3)	
**Age, years**							
Mean ±SD	59.0 ±9.3	58.8 ±9.0	59.0 ±9.2	58.8 ±9.2	59.2 ±9.4	59.4 ±9.5	0.002
**Body mass index, kg/m²**							
Mean ±SD	26.4 ±3.9	26.1 ±3.8	26.3 ±3.8	26.4 ±3.9	26.5 ±4.0	26.5 ±4.1	< 0.001
**Cigarette smoking [n (%)]**							
Current	2895 (11.7)	457 (9.2)	501 (10.1)	569 (11.4)	575 (11.7)	793 (16.1)	< 0.001
Former	10411 (42.0)	2033 (40.7)	2083 (42.1)	2044 (41.0)	2132 (43.4)	2119 (43.1)	
Never	11453 (46.3)	2502 (50.1)	2361 (47.7)	2378 (47.6)	2203 (44.9)	2009 (40.8)	
**Social class dichotomised [n (%)]**							
Non-manual	14691 (60.1)	3336 (67.4)	3170 (64.8)	2950 (59.8)	2840 (58.9)	2395 (49.5)	< 0.001
Manual	9741 (39.9)	1610 (32.6)	1722 (35.2)	1985 (40.2)	1982 (41.1)	2442 (50.5)	
**Level of education [n (%)]**							
Higher level	15841 (63.5)	3439 (68.5)	3373 (67.7)	3218 (63.9)	3084 (62.4)	2727 (54.8)	< 0.001
Lower level	9118 (36.5)	1584 (31.5)	1611 (32.3)	1819 (36.1)	1858 (37.6)	2246 (45.2)	
**Travel distance to hospital, km**							
Mean ±SD	20.4 ±13.1	16.5 ±11.3	20.6 ±12.1	22.0 ±12.2	25.2 ±13.2	17.5 ±14.5	< 0.001
**Travel time to hospital, minutes**							
Mean ±SD	20.8 ±10.3	18.0 ±8.9	20.8 ±9.5	21.9 ±9.4	24.4 ±10.6	19.0 ±11.6	< 0.001
**Urban or rural location [n (%)]**							
Urban	11214 (44.9)	2500 (49.8)	1832 (36.8)	1810 (35.9)	1575 (31.8)	3497 (70.2)	< 0.001
Rural	13763 (55.1)	2523 (50.2)	3153 (63.2)	3230 (64.1)	3373 (68.2)	1484 (29.8)	
**Moved house between 2000 and 2014 [n (%)]**							
Moved house	5355 (22.2)	963 (19.8)	972 (20.4)	1091 (22.4)	1060 (22.4)	1269 (26.1)	< 0.001
Did not move house	18728 (77.8)	3903 (80.2)	3799 (79.6)	3774 (77.6)	3662 (77.6)	3590 (73.9)	
**Deaths prior to March 2018 [n (%)]**							
Dead	8727 (35.0)	1630 (32.5)	1704 (34.3)	1703 (33.9)	1781 (36.1)	1909 (38.4)	< 0.001
Alive	16198 (65.0)	3386 (67.5)	3270 (65.7)	3327 (66.1)	3155 (63.9)	3060 (61.6)	
**Hospital activity 1999-2018**							
No admissions	2628 (10.5)	543 (10.8)	528 (10.6)	539 (10.7)	559 (11.3)	459 (9.2)	0.011
One or more admissions	22316 (89.5)	4476 (89.2)	4449 (89.4)	4494 (89.3)	4383 (88.7)	4514 (90.8)	
7 or more admissions	16497 (66.1)	3417 (68.1)	3295 (66.2)	3332 (66.2)	3291 (66.6)	3162 (63.6)	< 0.001
>20 hospital days	15144 (60.7)	3185 (63.5)	3054 (61.4)	3097 (61.5)	2959 (59.9)	2849 (57.3)	< 0.001
**Time spent in hospital 1999-2018, days**							
Full cohort 1999-2018, mean ±SD	32.8 ±63.0	30.5 ±54.9	33.0 ±69.0	31.2 ±57.2	32.5 ±62.9	37.0 ±69.6	< 0.001
Hospital attenders 1999-2018, mean ±SD	36.7 ±65.6	34.2 ±57.0	36.9 ±72.0	34.9 ±59.5	36.6 ±65.7	40.8 ±72.0	< 0.001
**Number of inpatient admissions 1999-2018**							
Full cohort 1999-2018, mean ±SD	7.5 ±26.0	7.3 ±22.8	8.2 ±36.1	7.6 ±30.8	6.7 ±11.7	7.6 ±21.4	0.073
Hospital attenders 1999-2018, mean ±SD	8.4 ±27.3	8.2 ±24.0	9.2 ±38.1	8.6 ±32.5	7.6 ±12.1	8.4 ±22.3	0.095

Round brackets in intervals denote strict inequalities (< or >); square brackets denote non-strict inequalities (≤ or ≥)

For peer review only

Table 2 shows the multivariable logistic regression for quintiles of Townsend Index and three outcomes: any hospital admissions, ≥7 hospital admissions, and >20 days of hospital stay between 1999 and 2018 Model 1 is adjusted for age and sex while model 2 is additionally adjusted for manual social class, lower education level, current cigarette smoking and body mass index >30 kg/m2. Additionally, each model is repeated in the subset of participants who survived to the end of the follow-up period. Compared to those with Townsend Deprivation Index quintiles 1 to 4 (lower than the average for England and Wales), those with a deprivation index in quintile 5 had a higher risk of spending more than 20 days in hospital multivariable adjusted odds ratio (OR) 1.18 (95% confidence interval (CI) 1.07–1.29) and for 7 or more hospital admissions OR 1.11 (95% CI 1.02–1.22), but there was no association for any hospital admission. The multivariable adjusted p-value for trend across quintiles of Townsend Index was 0.001 for more than 20 hospital days and 0.107 for 7 or more admissions. Associations in the subset of participants surviving to March 2018 (n=16 198) were higher than those for the full cohort.

Table 2 | Multivariable logistic regression of risk factors by quintiles of Townsend Index for any hospital admissions, ≥7 hospital admissions and >20 days of hospital stay from 1999 to 2018 in 24 977 men and women and in a subset of 16 198 men and women alive in March 2018)

	Quintile 1 [-6.74,-3.81]	Quintile 2 (-3.81,-2.94]	Quintile 3 (-2.94,-2.09]	Quintile 4 (-2.09,-0.64]	Quintile 5 (-0.64, 6.99]	p (trend)
**Outcome of any hospital admissions**						
Model 1	1.00	1.02 (0.89–1.16)	1.01 (0.89–1.15)	0.93 (0.82–1.05)	1.17 (1.02–1.34)	0.175
Model 1†	1.00	1.05 (0.91–1.21)	1.03 (0.89–1.18)	0.95 (0.83–1.09)	1.25 (1.08–1.45)	0.056
Model 2	1.00	1.00 (0.88–1.14)	1.00 (0.87–1.14)	0.90 (0.79–1.03)	1.09 (0.95–1.26)	0.731
Model 2†	1.00	1.04 (0.90–1.20)	1.01 (0.88–1.16)	0.91 (0.79–1.05)	1.18 (1.01–1.37)	0.341
**Outcome of 7 or more hospital admissions**						
Model 1	1.00	1.08 (0.99–1.18)	1.09 (1.00–1.18)	1.05 (0.97–1.15)	1.20 (1.10–1.31)	< 0.001
Model 1†	1.00	1.06 (0.95–1.19)	1.15 (1.03–1.29)	1.15 (1.03–1.29)	1.39 (1.25–1.56)	< 0.001
Model 2	1.00	1.07 (0.98–1.16)	1.05 (0.96–1.15)	1.01 (0.92–1.10)	1.11 (1.02–1.22)	0.107
Model 2†	1.00	1.05 (0.93–1.17)	1.11 (0.99–1.24)	1.09 (0.97–1.22)	1.28 (1.14–1.43)	< 0.001
**Outcome of more than 20 hospital days**						
Model 1	1.00	1.09 (1.00–1.19)	1.10 (1.00–1.20)	1.14 (1.04–1.24)	1.27 (1.17–1.39)	< 0.001
Model 1†	1.00	1.08 (0.96–1.22)	1.15 (1.02–1.30)	1.23 (1.09–1.40)	1.45 (1.28–1.64)	< 0.001
Model 2	1.00	1.08 (0.99–1.18)	1.05 (0.96–1.15)	1.09 (1.00–1.19)	1.18 (1.07–1.29)	0.001
Model 2†	1.00	1.06 (0.94–1.21)	1.09 (0.96–1.23)	1.16 (1.03–1.32)	1.34 (1.18–1.51)	< 0.001

Model 1: Adjusted for age and sex. Model 2: Adjusted for age, sex, manual social class, low education, current cigarette smoker, body mass index > 30kg/m2 † Excluding participants who died prior to April 2018

For peer review only

Figure 1 shows graphs of length of hospital stay by quintiles of Townsend index and demonstrates the disparity between individual socioeconomic factors and hospital stay when area deprivation index is also considered. In the first plot, results are stratified by higher and lower educational attainment. The difference in days between the least deprived (quintile 1) and the most deprived (quintile 5) is 6 days for those with lower educational attainment and 3 days for those with higher educational attainment. The second plot shows results stratified by manual and non-manual social class. The difference in days between the least deprived and the most deprived is 8 days for those with a manual social class and 3 days for those with a non-manual social class. Significant interactions were observed between social class, level of education and Townsend index ($p_{Interaction}$=0.025 and $p_{Interaction}$=0.020 respectively)

INSERT FIGURE 1 HERE

Supplementary table S1 shows the multivariable logistic regression for risk factors for outcomes of any hospital admissions, ≥7 hospital admissions and >20 days of hospital stay between 1999 and 2018 Models are presented for all participants, men and women and each risk factor is adjusted for all others for the nine models. Male sex is only included in the models for all participants. As well as age, social class, education and body mass index, the four individual components of Townsend index are modelled. As previously reported, age, male sex, lower education level, manual social class, current cigarette smoking and a body mass index >30 kg/m^2 were all associated with increased hospitalisation. No single component of the Townsend index was more strongly associated for all outcomes for both men and women. However, the unemployment component was associated with seven or more hospital admissions. Areas with low car ownership appeared to have a greater association in women than men.

Supplementary table S2 displays logistic regression models for the outcome of more than 20 hospital days for Townsend index in various subgroups. Models are stratified by a dichotomised subgroup: men and women, age above or below 65 years, manual and non-manual social class, lower or higher education level, smoking status, BMI above and below 30 kg/m^2, urban or rural home postcode, and moved house between the year 2000 and 2014. Odds ratios (OR) within all strata were in consistent directions with no interaction by age, smoking status or BMI.

The numbers of individuals with missing values for covariables were: 53 BMI, 218 smoking status, 545 social class, 18 education level.

Discussion

Residential area deprivation was associated with future hospital usage independently of individual sociodemographic factors in particular, age, sex, social class and education, as well as lifestyle factors smoking and body mass index in this cohort of middle-aged and older men and women. Study participants in the highest fifth of the Townsend Index - those living in the most deprived areas, at or below the national average, were more likely to spend more than 20 days in hospital or be admitted to hospital on more than 7 occasions. There were also significant interactions between residential area deprivation and individual social class and education level. Participants with a manual social class living in an area with higher deprivation index spent longer in hospital than those with manual occupations living in less deprived areas. Similarly, those with lower education level living in more deprived areas had the greatest risk of hospitalisation. This suggests that hospitalisation is greatest when those with poorer individual socioeconomic factors are combined with residential deprivation. We considered a number of possible explanations for these findings.

Strengths and limitations of this study

The EPIC-Norfolk cohort is very well characterised. This enabled us to take into account many potentially confounding variables understood to be related to hospital usage and disease. The UK National Health Service is free at the point of use and consequently income is not a major determinant of hospital admissions. Despite this,

For peer review only

social class, education and residential deprivation were all independently related to hospital use. Our study examines hospital activity using a prospective cohort design in a population of community-dwelling participants with clearly defined population denominators. It uses a large cohort of middle-aged and older men and women with 19 years of follow-up time having both area based census measures and individual social class and education level from questionnaires available.

Townsend Area Deprivation Index is associated with individual sociodemographic factors such as occupational social class and education and other factors including age, sex and BMI. Since all these factors are also related to hospital use, some level of confounding will be present. However, multivariable regression models adjusting for all these variables only modestly attenuated the area deprivation associations. In table S2, we stratified by the main confounders and the results remained consistent in the subgroups. The accuracy of the measurement might not be sufficient to ensure adequate adjustment, so we cannot exclude the possibility of residual confounding with known or other unknown factors associated with both Townsend Index and hospital usage. These unknown factors may either attenuate or strengthen the associations. Interactions between area deprivation and individual sociodemographic factors highlighted stronger associations among more deprived groups.

The use of area-based measurements has some limitations. The factors used in the Townsend score may vary in their ability to assess deprivation according to setting. In urban areas, lower car ownership rates may reflect the availability of other transport options and closer proximity of work places and facilities such as shops. In rural areas, overcrowding may be less common while car ownership may be more of a necessity while simultaneously a drain on resources. The deprivation index is based on data from the UK census that only takes place every ten years and over the period under examination, areas may change becoming more or less deprived.

Area deprivation was determined by postcode of residence in the year 2000. Study participants who moved house may have been misclassified for some of the period over which hospitalisation was assessed. However, while 22% of the cohort moved house between the years 2000 and 2014, the large majority of participants relocated locally in Norfolk, with others moving elsewhere in England and Wales. Since the Townsend Index was not measured at enumeration district level in the UK census beyond 1991, no directly comparable measure was available at later time points to examine change. However, a sensitivity analysis of non-movers found very similar results to the main analyses and any misclassification due to moves or changes over time in residential area deprivation scoring and resultant measurement error would only be likely to attenuate associations with the residential area score. HES record were available for participants who relocated within England and Wales and hence there was virtually no loss to follow-up.

Differential misclassification in hospital use may be explained by early death rates. Study participants living in more deprived areas may have died earlier and not used hospital services for the full period. However, while the death rate was higher among those living in the most deprived areas, 65% of the cohort survived beyond 2018 and models restricted to survivors were more strongly associated with outcome measures than those in the main analysis. Sociodemographic factors may be less relevant for the very seriously ill who require hospital treatment at the end of life.

It may also be possible that individuals did not use NHS facilities but private hospitals differently by socioeconomic status which might explain lower use in the higher sociodemographic groups. However, the use of private hospitals in the Norfolk area over this time period was minimal [27] and hence record linkage of routinely collected hospital episode data gave virtually complete ascertainment. Reverse causation is also possible whereby those in poor health at recruitment may have lower occupational social class increasing the chance of them living in a more deprived area. However, hospitalisation rates were low in the period directly after recruitment.

Comparison with other studies

Inequality in healthcare favouring the better off has been observed in many countries [10–12] and healthcare insurance and eligibility for government healthcare based on income thresholds may influence the associations

For peer review only

observed. NHS healthcare is not constrained by ability to pay and hence we were able to examine the independent association of residential area deprivation - material living conditions, poor quality housing and poor infrastructure - and its association with subsequent hospitalisation.

There is some evidence to suggest that travel time is associated with hospital use [31,32] but there was no strong association in this study. Study participants were approximately evenly divided into those living in urban and those in rural areas. The moderately deprived (those with Townsend quintile 2-4) were more likely to live in rural areas while the most deprived (Townsend quintile 5) were predominantly urban dwellers. Study participants in Townsend quintiles 1 and 5 were closer by road from their home to the Norfolk and Norwich hospital but the time taken for the journey did not vary greatly. Neither distance from hospital nor urban or rural location explained our findings, since those in the lowest deprivation areas are mainly urban with the shortest travel time to hospital. Studies examining urban/rural populations and car ownership have noted differences in deprivation characteristics. [33,34] However, irrespective of travel distance or time, owning or having access to a car would make a considerable difference in being able to access local facilities. Although there may be more regular public transport services in cities, this will vary and cost and limited travel options may restrict access not only to hospital but also to friends and relatives, to better quality supermarkets and to parks and recreational facilities.

Most studies examining deprivation in the context of health, disease and mortality either rely on area based measures collected for example from census data [16–20] or from individual level data from questionnaires. [13,14] We had access to both forms of information, having derived individual social class and education level from self reported questionnaires and area level measures from residential postcode linkage. Hospital based studies using patients as study participants do not have a reliable population based denominator and cannot estimate overall risk in the population. Studies often attempt to define a denominator using separate population estimates while not individually linking. [16,35,36] We were able to examine hospital usage over 19 years in a clearly defined community based population using a prospective cohort design.

Norfolk is an area of generally low deprivation with >80% of the study population living in areas with deprivation levels below the national average. Few participants live in areas of high deprivation such as those found in some larger cities in other parts of the country. Those living in more deprived cities or regions have a socioeconomic gradient in hospital usage more extreme than we were able to observe [37] but while our study does not provide any information on the most extreme forms of deprivation, there was sufficient heterogeneity to observe large differences in hospital use.

Our results provide further evidence adding to the substantial literature linking deprivation to health. Unlike many studies, we used overall measures of hospital activity, including both elective and emergency admissions and found evidence of an independent association of residential area deprivation not accounted for by known individual factors such as social class and education. Our results also demonstrate that the combination of residential area deprivation with lower levels of education or manual social class result in the highest levels of hospitalisation.

The Black report [2,3] concluded that health inequalities were not mainly attributable to failings in the NHS, but rather to many other social inequalities influencing health: income, education, housing, diet, employment, and conditions of work. It suggested two mechanisms for how social determinants influence health: cultural/behavioural and materialist/structuralist. Some authors have pointed out that research on the determinants of health are generally focused on the individual but patterns of population health are unclear without examining structural determinants at the societal level. [38] Townsend's residential deprivation index uses aggregate measures of particular characteristics for people living in an area. It has been used mainly as a surrogate for individual measures of deprivation in many studies. [39] We were not able to examine physical features of the environment in this study. Ecological measurements such as the quality of housing, access to recreational facilities, local services provided, community support and levels of crime may affect health and hospital usage. However, we were able to examine both individual and area level deprivation in the same study participants, and the interaction we observed suggests that there is a higher risk hospitalisation in more deprived

For peer review only

areas of residence disproportionately for those with lower individual social class and education. Conversely, individuals with non-manual social class and higher levels of education appear more resilient to hospitalisation irrespective of the level of deprivation of their residence.

Conclusions and policy implications

There is a socioeconomic gradient in hospital usage for factors measured both individually and at area level. Residential area deprivation predicts future hospitalisations, time spent in hospital and number of admissions, independently of individual social class and education level and other behavioural factors. There are significant interactions such that residential area deprivation has greater impact in those with low education level or manual social class. Conversely, higher education level and social class mitigated the association of area deprivation with hospital usage. Effective NHS and government policy should therefore involve addressing deprivation both at the individual and infrastructural levels to identify and target those most at risk within the community. NHS policies focused on reducing health inequalities in the elderly need to work alongside wider government initiatives to improve the quality of housing, transport and infrastructure and access to recreation and green space.

References

1 Antonovsky A. Social Class and Illness: A Reconsideration. *Sociological Inquiry* 1967;**37**:311–22. doi:10.1111/j.1475-682X.1967.tb00661.x

2 Black D. Inequalities in Health: Report of a Research Working Group. 1980.

3 Acheson D. *Independent inquiry into inequalities in health: report*. HM Stationery Office 1998.

4 Smith GD, Bartley M, Blane D. The Black report on socioeconomic inequalities in health 10 years on. *BMJ* 1990;**301**:373–7.

5 Health expectancies at birth and at age 65 in the UK, based on 2011 Census health and disability prevalence data - Office for National Statistics. https://www.ons.gov.uk/peoplepopulationandcommunity/healthandsocialcare/healthandlifeexpectancies/bulletins/healthexpectanciesatbirthandatage65intheukbasedon2011censushealthanddisabilityprevalencedata/2010to2012 (accessed 23 May 2018).

6 Life Expectancy at Birth and at Age 65 by Local Areas in England and Wales - Office for National Statistics. https://www.ons.gov.uk/peoplepopulationandcommunity/birthsdeathsandmarriages/lifeexpectancies/bulletins/lifeexpectancyatbirthandatage65bylocalareasinenglandandwales/2015-11-04 (accessed 23 May 2018).

7 Marmot M, Friel S, Bell R, *et al.* Closing the gap in a generation: health equity through action on the social determinants of health. *The Lancet* 2008;**372**:1661–9. doi:10.1016/S0140-6736(08)61690-6

8 Stafford M, Marmot M. Neighbourhood deprivation and health: does it affect us all equally? *Int J Epidemiol* 2003;**32**:357–66. doi:10.1093/ije/dyg084

9 Townsend P, Phillimore P, Beattie A. Health and deprivation. Inequality and the North. *Croom Helm Ltd, London* Published Online First: 1987. doi:10.1016/0168-8510(88)90006-1

10 Doorslaer E van, Masseria C, Koolman X. Inequalities in access to medical care by income in developed countries. *CMAJ* 2006;**174**:177–83. doi:10.1503/cmaj.050584

11 Pickett KE, Wilkinson RG. Income inequality and health: A causal review. *Social Science & Medicine* 2015;**128**:316–26. doi:10.1016/j.socscimed.2014.12.031

12 Fjær EL, Balaj M, Stornes P, *et al.* Exploring the differences in general practitioner and health care specialist utilization according to education, occupation, income and social networks across Europe: findings from the

For peer review only - http://bmjopen.bmj.com/site/about/guidelines.xhtml

European social survey (2014) special module on the social determinants of health. *Eur J Public Health* 2017;**27**:73–81. doi:10.1093/eurpub/ckw255

13 Head J, Chungkham HS, Hyde M, *et al.* Socioeconomic differences in healthy and disease-free life expectancy between ages 50 and 75: a multi-cohort study. *Eur J Public Health* 2019;**29**:267–72. doi:10.1093/eurpub/cky215

14 McFadden E, Luben R, Wareham N, *et al.* Occupational social class, risk factors and cardiovascular disease incidence in men and women: a prospective study in the European Prospective Investigation of Cancer and Nutrition in Norfolk (EPIC-Norfolk) cohort. *Eur J Epidemiol* 2008;**23**:449. doi:10.1007/s10654-008-9262-2

15 Luben R, Hayat S, Wareham N, *et al.* Predicting admissions and time spent in hospital over a decade in a population-based record linkage study: the EPIC-Norfolk cohort. *BMJ Open* 2016;**6**:e009461. doi:10.1136/bmjopen-2015-009461

16 Asaria M, Doran T, Cookson R. The costs of inequality: whole-population modelling study of lifetime inpatient hospital costs in the English National Health Service by level of neighbourhood deprivation. *J Epidemiol Community Health* 2016;**70**:990–6. doi:10.1136/jech-2016-207447

17 Bernard S, Smith LK. Emergency admissions of older people to hospital: a link with material deprivation. *J Public Health (Oxf)* 1998;**20**:97–101. doi:10.1093/oxfordjournals.pubmed.a024727

18 Conway R, Galvin S, Coveney S, *et al.* Deprivation as an outcome determinant in emergency medical admissions. *QJM* 2013;**106**:245–51. doi:10.1093/qjmed/hcs233

19 Conway R, Byrne D, O'Riordan D, *et al.* Deprivation index and dependency ratio are key determinants of emergency medical admission rates. *European Journal of Internal Medicine* 2015;**26**:709–13. doi:10.1016/j.ejim.2015.09.010

20 Moore L, Cisse B, Batomen Kuimi BL, *et al.* Impact of socio-economic status on hospital length of stay following injury: a multicenter cohort study. *BMC Health Serv Res* 2015;**15**. doi:10.1186/s12913-015-0949-2

21 Macintyre S, Maciver S, Sooman A. Area, Class and Health: Should we be Focusing on Places or People? *Journal of Social Policy* 1993;**22**:213. doi:10.1017/S0047279400019310

22 Smith GD, Hart C, Watt G, *et al.* Individual social class, area-based deprivation, cardiovascular disease risk factors, and mortality: the Renfrew and Paisley Study. *J Epidemiol Community Health* 1998;**52**:399–405.

23 Shohaimi S, Luben R, Wareham N, *et al.* Residential area deprivation predicts smoking habit independently of individual educational level and occupational social class. A cross sectional study in the Norfolk cohort of the European Investigation into Cancer (EPIC-Norfolk). *Journal of Epidemiology & Community Health* 2003;**57**:270–6. doi:10.1136/jech.57.4.270

24 Hayat SA, Luben R, Keevil VL, *et al.* Cohort Profile: A prospective cohort study of objective physical and cognitive capability and visual health in an ageing population of men and women in Norfolk (EPIC-Norfolk 3). *Int J Epidemiol* 2014;**43**:1063–72. doi:10.1093/ije/dyt086

25 Day N, Oakes S, Luben R, *et al.* EPIC-Norfolk: study design and characteristics of the cohort. European Prospective Investigation of Cancer. *Br J Cancer* 1999;**80 Suppl 1**:95–103.

26 Phillimore P, Beattie A, Townsend P. Widening inequality of health in northern England, 1981-91. *BMJ* 1994;**308**:1125–8.

27 Boyle S. United Kingdom (England): Health system review. *Health Syst Transit* 2011;**13**:1–483, xix–xx.

28 Bland R. Measuring "Social Class": A Discussion of the Registrar-General's Classification. *Sociology* 1979;**13**:283–91. doi:10.1177/003803857901300209

29 McFadden E, Luben R, Khaw K-T. Different measures of social class in women and mortality. *Eur J Epidemiol* 2009;**24**:231–6. doi:10.1007/s10654-009-9332-0

For peer review only

30 Luxen D, Vetter C. Real-time routing with OpenStreetMap data. In: *Proceedings of the 19th ACM SIGSPATIAL International Conference on Advances in Geographic Information Systems*. Chicago, Illinois: : ACM 2011. 513–6. doi:10.1145/2093973.2094062

31 Kelly C, Hulme C, Farragher T, *et al.* Are differences in travel time or distance to healthcare for adults in global north countries associated with an impact on health outcomes? A systematic review. *BMJ Open* 2016;**6**:e013059. doi:10.1136/bmjopen-2016-013059

32 Nicholl J, West J, Goodacre S, *et al.* The relationship between distance to hospital and patient mortality in emergencies: an observational study. *Emerg Med J* 2007;**24**:665–8. doi:10.1136/emj.2007.047654

33 Gilthorpe MS, Wilson RC. Rural/urban differences in the association between deprivation and healthcare utilisation. *Social Science & Medicine* 2003;**57**:2055–63. doi:10.1016/S0277-9536(03)00071-6

34 Christie SML, Fone DL. Does car ownership reflect socio-economic disadvantage in rural areas? A cross-sectional geographical study in Wales, UK. *Public Health* 2003;**117**:112–6. doi:10.1016/S0033-3506(02)00027-6

35 Billings J, Georghiou T, Blunt I, *et al.* Choosing a model to predict hospital admission: an observational study of new variants of predictive models for case finding. *BMJ Open* 2013;**3**:e003352. doi:10.1136/bmjopen-2013-003352

36 Gray DP, Henley W, Chenore T, *et al.* What is the relationship between age and deprivation in influencing emergency hospital admissions? A model using data from a defined, comprehensive, all-age cohort in East Devon, UK. *BMJ Open* 2017;**7**:e014045. doi:10.1136/bmjopen-2016-014045

37 Macintyre S, Macdonald L, Ellaway A. Do poorer people have poorer access to local resources and facilities? The distribution of local resources by area deprivation in Glasgow, Scotland. *Social Science & Medicine* 2008;**67**:900–14. doi:10.1016/j.socscimed.2008.05.029

38 Lynch JW, Kaplan GA. Understanding How Inequality in the Distribution of Income Affects Health. *J Health Psychol* 1997;**2**:297–314. doi:10.1177/135910539700200303

39 Macintyre S, Maciver S, Sooman A. Area, Class and Health: Should we be Focusing on Places or People? *Journal of Social Policy* 1993;**22**:213–34. doi:10.1017/S0047279400019310

For peer review only

Supporting information

Supplementary Table S1 | Multivariable logistic regression of risk factors for any hospital admissions, ≥7 hospital admissions and >20 days of hospital stay from 1999 to 2018 in 24 977 men and women. Townsend index components using the UK 1991 census

Supplementary Table S2 | Multivariable logistic regression of Townsend Index and more than twenty hospital days in subgroups

Figure 1: Hospitalisation by deprivation index. Length of hospital stay over 19 years of follow-up by quintiles of Townsend Index grouped by categories of education level and categories of social class. Low education level is defined as those having no qualifications and high education as those with at least some qualifications at secondary level or above. Length of stay is truncated to 365 days for those staying longer than 365 days. Interaction tested using multivariable adjusted linear regression with covariables age, sex, education level (higher/lower), body mass index (≤30/>30) smoking status (current/non-current)

For peer review only

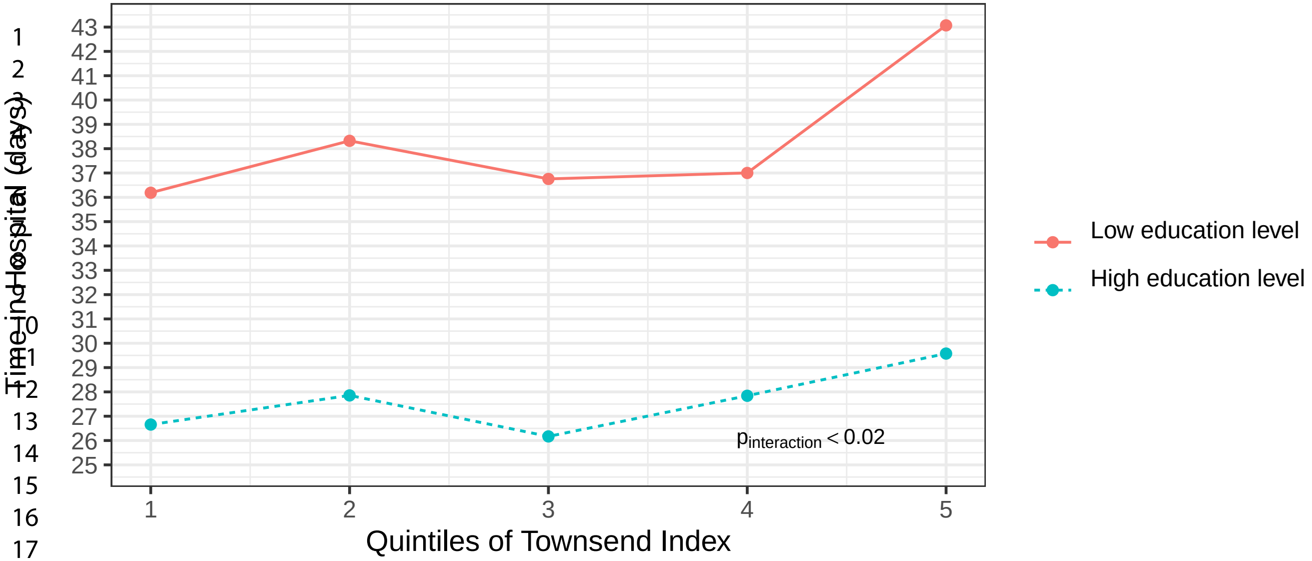
Time in Hospital (days)
Quintiles of Townsend Index
Low education level
High education level
$p_{interaction} < 0.02$

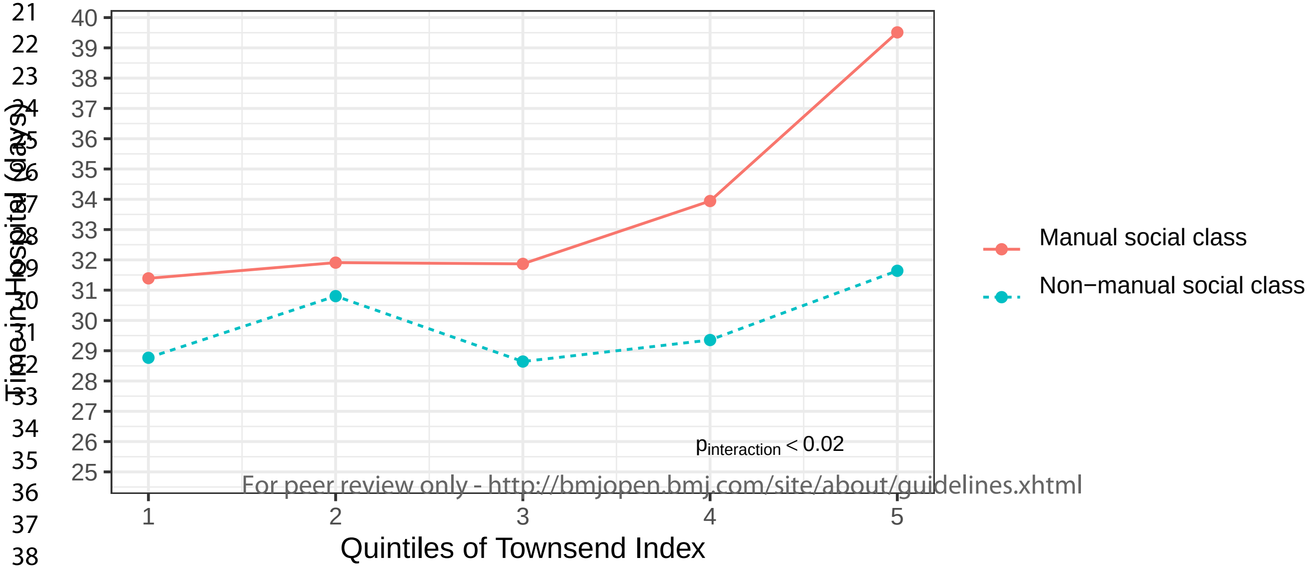
Time in Hospital (days)
Quintiles of Townsend Index
Manual social class
Non−manual social class
$p_{interaction} < 0.02$

Supplementary Table S1 | Multivariable logistic regression of risk factors for any hospital admissions, ≥7 hospital admissions and >20 days of hospital stay from 1999 to 2018 in 24 977 men and women. Townsend index components using the UK 1991 census

	All subjects OR (95% CI)	p value	Men OR (95% CI)	p value	Women OR (95% CI)	p value
**Outcome of any hospital admissions**						
Male sex	1.13 (1.04–1.23)	0.006				
Age per 10 years	2.08 (1.98–2.20)	< 0.001	2.15 (1.99–2.34)	< 0.001	2.03 (1.89–2.17)	< 0.001
Unemployment Z-Score >0	1.06 (0.92–1.21)	0.426	1.06 (0.87–1.31)	0.560	1.05 (0.88–1.26)	0.604
Households with no car Z-Score >0	1.05 (0.89–1.23)	0.568	0.91 (0.72–1.16)	0.459	1.16 (0.94–1.44)	0.161
Households not owner-occupied Z-Score >0	1.03 (0.90–1.19)	0.625	0.99 (0.81–1.22)	0.948	1.07 (0.89–1.28)	0.479
Household overcrowding Z-Score >0	0.98 (0.88–1.10)	0.760	1.00 (0.84–1.19)	0.983	0.97 (0.84–1.13)	0.717
Manual social class	1.16 (1.06–1.27)	0.001	1.25 (1.08–1.43)	0.002	1.11 (0.98–1.25)	0.106
Low education level	1.35 (1.22–1.50)	< 0.001	1.21 (1.03–1.43)	0.026	1.45 (1.27–1.66)	< 0.001
Current smoker	1.21 (1.06–1.38)	0.006	1.15 (0.94–1.41)	0.174	1.26 (1.05–1.51)	0.013
BMI>30 kg/m^2	1.28 (1.12–1.46)	< 0.001	1.19 (0.97–1.47)	0.095	1.34 (1.13–1.59)	< 0.001
**Outcome of 7 or more hospital admissions**						
Male sex	1.23 (1.16–1.30)	< 0.001				
Age per 10 years	1.49 (1.44–1.54)	< 0.001	1.50 (1.43–1.57)	< 0.001	1.48 (1.42–1.55)	< 0.001
Unemployment Z-Score >0	1.18 (1.08–1.28)	< 0.001	1.17 (1.04–1.33)	0.012	1.18 (1.06–1.33)	0.004
Households with no car Z-Score >0	0.98 (0.88–1.08)	0.641	0.92 (0.79–1.06)	0.253	1.03 (0.90–1.17)	0.692
Households not owner-occupied Z-Score >0	0.93 (0.86–1.02)	0.134	0.91 (0.80–1.04)	0.163	0.95 (0.85–1.08)	0.447
Household overcrowding Z-Score >0	1.08 (1.00–1.16)	0.044	1.07 (0.96–1.19)	0.207	1.08 (0.98–1.20)	0.112
Manual social class	1.15 (1.08–1.22)	< 0.001	1.16 (1.06–1.26)	< 0.001	1.14 (1.06–1.24)	0.001
Low education level	1.14 (1.07–1.21)	< 0.001	1.12 (1.02–1.23)	0.014	1.15 (1.06–1.25)	< 0.001
Current smoker	1.34 (1.23–1.46)	< 0.001	1.25 (1.10–1.41)	< 0.001	1.44 (1.28–1.61)	< 0.001
BMI>30 kg/m^2	1.38 (1.28–1.49)	< 0.001	1.39 (1.24–1.56)	< 0.001	1.38 (1.25–1.52)	< 0.001
**Outcome of more than 20 hospital days**						
Male sex	1.16 (1.10–1.23)	< 0.001				
Age per 10 years	2.68 (2.59–2.78)	< 0.001	2.62 (2.49–2.76)	< 0.001	2.73 (2.61–2.87)	< 0.001
Unemployment Z-Score >0	1.10 (1.01–1.20)	0.034	1.10 (0.96–1.25)	0.161	1.10 (0.98–1.24)	0.111
Households with no car Z-Score >0	1.00 (0.90–1.11)	0.979	0.95 (0.81–1.11)	0.501	1.04 (0.90–1.19)	0.599
Households not owner-occupied Z-Score >0	1.00 (0.92–1.10)	0.916	1.01 (0.88–1.16)	0.849	1.00 (0.88–1.13)	0.958
Household overcrowding Z-Score >0	1.07 (0.99–1.15)	0.100	1.03 (0.92–1.15)	0.622	1.10 (0.99–1.22)	0.071
Manual social class	1.16 (1.09–1.24)	< 0.001	1.19 (1.09–1.31)	< 0.001	1.14 (1.05–1.24)	0.003
Low education level	1.16 (1.09–1.24)	< 0.001	1.18 (1.07–1.30)	< 0.001	1.15 (1.05–1.25)	0.001
Current smoker	1.57 (1.44–1.72)	< 0.001	1.61 (1.41–1.83)	< 0.001	1.54 (1.37–1.74)	< 0.001
BMI>30 kg/m^2	1.61 (1.49–1.74)	< 0.001	1.58 (1.40–1.79)	< 0.001	1.63 (1.48–1.81)	< 0.001

Supplementary Table S2 | Multivariable logistic regression of Townsend Index and more than twenty hospital days in subgroups

	Townsend Index OR (95% CI)†
**Men and women**	
Men (n=11214)	1.01 (0.99–1.03)
Women (n=13763)	1.03 (1.01–1.05)
**By age above and below 65 years**	
Less than 65 years (n=17343)	1.03 (1.01–1.05)
65 years and above (n=7634)	1.01 (0.98–1.03)
**Manual and non-manual social class**	
Non-manual (n=14691)	1.02 (1.00–1.04)
Manual (n=9741)	1.03 (1.00–1.05)
**By level of education**	
Higher level (n=15841)	1.01 (0.99–1.03)
Lower level (n=9118)	1.03 (1.01–1.06)
**By smoking status**	
Former or never smoker (n=21864)	1.02 (1.01–1.04)
Current smoker (n=2895)	1.00 (0.97–1.04)
**By level of body mass index**	
BMI ≤ 30 kg/m² (n=21124)	1.02 (1.01–1.04)
BMI > 30 kg/m² (n=3800)	1.01 (0.98–1.04)
**Urban or rural home postcode**	
Urban (n=11214)	1.01 (1.00–1.03)
Rural (n=13763)	1.03 (1.01–1.06)
**Moved house between 2000 and 2014**	
Moved house (n=5355)	1.04 (1.01–1.07)
Did not move house (n=18728)	1.01 (1.00–1.03)

Logistic regression per unit Townsend Index. † Adjusted for age, current smoking, BMI (categories)

STROBE Statement—Checklist of items that should be included in reports of ***cohort studies***

	Item No	Recommendation	Page No
**Title and abstract**	1	(*a*) Indicate the study's design with a commonly used term in the title or the abstract	1
		(*b*) Provide in the abstract an informative and balanced summary of what was done and what was found	2
**Introduction**			
Background/rationale	2	Explain the scientific background and rationale for the investigation being reported	4
Objectives	3	State specific objectives, including any prespecified hypotheses	4
**Methods**			
Study design	4	Present key elements of study design early in the paper	5 and title
Setting	5	Describe the setting, locations, and relevant dates, including periods of recruitment, exposure, follow-up, and data collection	5
Participants	6	(*a*) Give the eligibility criteria, and the sources and methods of selection of participants. Describe methods of follow-up	6
		(*b*) For matched studies, give matching criteria and number of exposed and unexposed	
Variables	7	Clearly define all outcomes, exposures, predictors, potential confounders, and effect modifiers. Give diagnostic criteria, if applicable	6
Data sources/ measurement	8*	For each variable of interest, give sources of data and details of methods of assessment (measurement). Describe comparability of assessment methods if there is more than one group	5-6
Bias	9	Describe any efforts to address potential sources of bias	10-11
Study size	10	Explain how the study size was arrived at	6
Quantitative variables	11	Explain how quantitative variables were handled in the analyses. If applicable, describe which groupings were chosen and why	6
Statistical methods	12	(*a*) Describe all statistical methods, including those used to control for confounding	6
		(*b*) Describe any methods used to examine subgroups and interactions	Table S2

For peer review only - http://bmjopen.bmj.com/site/about/guidelines.xhtml

		(*c*) Explain how missing data were addressed	6
		(*d*) If applicable, explain how loss to follow-up was addressed	11
		(*e*) Describe any sensitivity analyses	10
**Results**			
Participants	13*	(a) Report numbers of individuals at each stage of study—eg numbers potentially eligible, examined for eligibility, confirmed eligible, included in the study, completing follow-up, and analysed	5-6
		(b) Give reasons for non-participation at each stage	6
		(c) Consider use of a flow diagram	
Descriptive data	14*	(a) Give characteristics of study participants (eg demographic, clinical, social) and information on exposures and potential confounders	Table 1
		(b) Indicate number of participants with missing data for each variable of interest	9
		(c) Summarise follow-up time (eg, average and total amount)	Stated in title
Outcome data	15*	Report numbers of outcome events or summary measures over time	Table 1

For peer review only - http://bmjopen.bmj.com/site/about/guidelines.xhtml

Main results	16	(*a*) Give unadjusted estimates and, if applicable, confounder-adjusted estimates and their precision (eg, 95% confidence interval). Make clear which confounders were adjusted for and why they were included	8, table 2
		(*b*) Report category boundaries when continuous variables were categorized	6 (Statistical analysis)
		(*c*) If relevant, consider translating estimates of relative risk into absolute risk for a meaningful time period	
Other analyses	17	Report other analyses done—eg analyses of subgroups and interactions, and sensitivity analyses	9, Table S2, Covariables in subgroups
**Discussion**			
Key results	18	Summarise key results with reference to study objectives	10
Limitations	19	Discuss limitations of the study, taking into account sources of potential bias or imprecision. Discuss both direction and magnitude of any potential bias	10-11
Interpretation	20	Give a cautious overall interpretation of results considering objectives, limitations, multiplicity of analyses, results from similar studies, and other relevant evidence	12
Generalisability	21	Discuss the generalisability (external validity) of the study results	12
**Other information**			
Funding	22	Give the source of funding and the role of the funders for the present study and, if applicable, for the original study on which the present article is based	

*Give information separately for exposed and unexposed groups.

Note: An Explanation and Elaboration article discusses each checklist item and gives methodological background and published examples of transparent reporting. The STROBE checklist is best used in conjunction with this article (freely available on the Web sites of PLoS Medicine at http://www.plosmedicine.org/, Annals of Internal Medicine at http://www.annals.org/, and Epidemiology at http://www.epidem.com/). Information on the STROBE Initiative is available at http://www.strobe-statement.org.
